# High-Efficiency Targeted Editing of Large Viral Genomes by RNA-Guided Nucleases

**DOI:** 10.1371/journal.ppat.1004090

**Published:** 2014-05-01

**Authors:** Yanwei Bi, Le Sun, Dandan Gao, Chen Ding, Zhihua Li, Yadong Li, Wei Cun, Qihan Li

**Affiliations:** 1 Institute of Medical Biology, Chinese Academy of Medical Sciences & Peking Union Medical College, Kunming, Yunnan, China; 2 Yunnan Key Laboratory of Vaccine Research and Development of Severe Infectious Disease, Kunming, Yunnan, China; University of Southern California Keck School of Medicine, United States of America

## Abstract

A facile and efficient method for the precise editing of large viral genomes is required for the selection of attenuated vaccine strains and the construction of gene therapy vectors. The type II prokaryotic CRISPR-Cas (clustered regularly interspaced short palindromic repeats (CRISPR)-associated (Cas)) RNA-guided nuclease system can be introduced into host cells during viral replication. The CRISPR-Cas9 system robustly stimulates targeted double-stranded breaks in the genomes of DNA viruses, where the non-homologous end joining (NHEJ) and homology-directed repair (HDR) pathways can be exploited to introduce site-specific indels or insert heterologous genes with high frequency. Furthermore, CRISPR-Cas9 can specifically inhibit the replication of the original virus, thereby significantly increasing the abundance of the recombinant virus among progeny virus. As a result, purified recombinant virus can be obtained with only a single round of selection. In this study, we used recombinant adenovirus and type I herpes simplex virus as examples to demonstrate that the CRISPR-Cas9 system is a valuable tool for editing the genomes of large DNA viruses.

## Author Summary

The clustered regularly interspaced short palindromic repeats (CRISPR)-associated (Cas) system was discovered as a component of the bacterial acquired immune system that cleaves foreign DNA. This system is now used for site-specific genome editing in a wide range of organisms, including bacteria, yeasts, plants, and animals. However, the use of this approach in non-cell organisms, such as non-integrating viruses, has not been reported. Because multiple steps are required to construct mutant or recombinant DNA viruses with large genomes using the current approaches, we used the CRISPR-Cas9 system to introduce site-specific indels and insert a foreign gene into an adenoviral vector and wild-type herpes simplex virus. The high efficiency of CRISPR-Cas9 editing allowed for simple construction and purification of recombinant progeny virus. We believe that this new technique will have broad practical significance for selecting attenuated vaccine strains and antiviral drugs, constructing gene therapy vectors, and establishing efficient methods for viral biological studies.

## Introduction

With the rapid development of biotechnology, site-specific genome editing approaches allow researchers to target any gene within any organism. Two well-known genome-editing technologies include zinc-finger nucleases (ZFNs) and transcription activator-like effector nucleases (TALENs). These approaches function by using a nuclease to specifically target a gene and cleave its DNA to induce double-stranded breaks (DSBs) at the target site. The breakage then triggers the cellular DNA repair mechanisms, including error-prone non-homologous end joining (NHEJ) and homology-directed repair (HDR) [1]. However, customizing gene disruption using either ZFNs or TALENs requires the design of specific proteins to target each dsDNA site [2], [3].

Clustered regularly interspaced short palindromic repeats (CRISPR)- CRISPR-associated 9 (Cas9) is a recently discovered, site-specific genome editing system that is part of the CRISPR-Cas bacterial acquired immune system, which cleaves foreign DNA [4], [5], [6]. The Cas9 protein belongs to the type II CRISPR-Cas system that, with the guidance of a CRISPR RNA (crRNA) and trans-activating crRNA (tracrRNA), cleaves DNA matching the crRNA in a sequence-specific manner [7], [8]. Moreover, a recent study by Jinek et al. demonstrated that the crRNA-tracrRNA complex can be fused to form guide RNAs (gRNAs) that function in specific DNA recognition and Cas9 protein binding [8]. The CRISPR-Cas9 system requires the design of only a single guide sequence that matches the DNA targeted for cleavage. This property greatly increases the system's ease of use compared with ZFN and TALEN-based genome editing. Since the first report of the use of CRISPR-Cas9 for genome editing in human cells in early 2013 [9], [10], [11], [12], this technology has been used *in vivo* in human cells and other organisms, including *Streptococcus pneumoniae*, *Escherichia coli*, *Saccharomyces cerevisiae*, crop plants, *Arabidopsis*, *Nicotiana benthamiana*, *Danio rerio* (zebrafish), mice, and rats [13], [14], [15], [16], [17], [18], [19], [20], [21], [22], [23]. Nevertheless, as mentioned in several recent publications, Cas9 tolerates mismatches between the guide RNA and target DNA in a sequence-dependent manner and potentially tolerates up to five target mismatches in human cells [12], [24], [25], [26], [27], thereby promoting undesired off-target mutations. To improve the specificity of Cas9-mediated genome editing, a strategy that combines a mutant nickase version of Cas9 (Cas9n) with a pair of offset gRNAs that bind to opposite DNA strands at the target locus was developed. In cell lines, this strategy reduces off-target activity by 50- to 1500-fold without sacrificing cleavage efficiency [28].

Non-cellular microorganisms such as viruses replicate within the living cells of other organisms and can infect all forms of life, thus causing diseases. These organisms are also potential gene therapy vectors. The sizes of viral genomes vary greatly, and their genetic information can range from 1 Kb to 2.47 Mb [29]. Based on the accessibility of genetic manipulation handling, those viruses with genome sizes less than 30 Kb, including DNA viruses such as Hepadnaviridae, Polyomaviridae, and Papillomaviridae and all RNA viruses, are categorized as small viral genomes. Normally, several monoclonal restriction enzyme digestion sites exist on a viral genome or cDNA, which makes mutant construction and fragment substitution easier. However, for those viruses with large viral genomes, including Adenoviridae, Herpesviridae, and Poxviridae, which have genome sizes larger than 30 Kb, monoclonal restriction enzyme digestion sites are not readily available, making the enzyme digestion and ligation processes more difficult. Currently available genome editing approaches use large fragment cloning procedures, such as two-step counter-selection and bacterial artificial chromosome (BAC) system construction. These methods require multiple steps, including continuous growth of transformants and the selection and construction of a BAC vector, which are time-consuming and labor-intensive [30], [31]. Therefore, it would be beneficial to establish a more efficient and straightforward genome editing technology for constructing mutant or recombinant large DNA viruses.

Ebina *et al.* used the CRISPR-Cas9 system to disrupt the latent HIV-1 provirus [32]. However, use of the CRISPR-Cas9 system to induce mutations in non-integrating viral genomes has not been reported, likely due to the genomic differences between viruses and cells. The genomes of viruses are much smaller than those of mammalian cells; therefore, cleavage of a DNA sequence in a virus is more likely to affect viral replication than mammalian cell replication. Furthermore, unlike mammalian cells [33], the genomes of DNA viruses are in a dissociated form, so DSBs may terminate viral replication.

We first confirmed the effectiveness of the CRISPR-Cas9 system at altering large-genome DNA viruses by introducing site-specific indels and inserting a foreign gene into an adenoviral vector (ADV) and type 1 herpes simplex virus (HSV1) in a single step using the CRISPR-Cas9 system. We found that this system interfered with viral replication and that the efficiency of genome mutation and recombination increased significantly. Our work demonstrates the versatility of the CRISPR-Cas9 platform for non-cellular microorganisms in an episomal form.

## Results

### Heritable and efficient targeted recombinant adenoviral genome editing by RNA-guided nucleases (RGNs)

**Figure 1:**
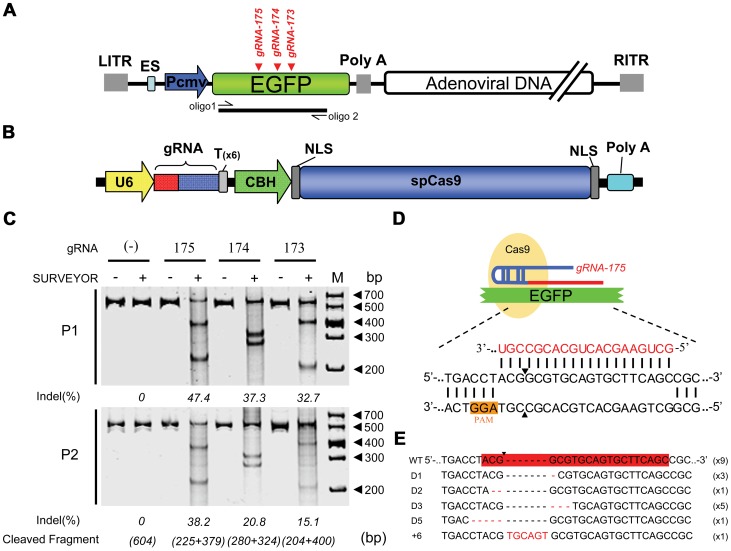
Cas9-mediated inheritable indels at specific sites in recombinant adenoviral genomes. (A) Schematic showing the recombinant adenoviral genome-harbored *EGFP* (ADV-EGFP), with the target sites of the guide RNAs (gRNA-175, gRNA-174, and gRNA-173) labeled in red. The fragment amplified by oligo 1 and oligo 2 was used in the SURVEYOR assays. (B) Construction of the bicistronic vector co-expressing SpCas9 and gRNA. (C) Top: SURVEYOR assay of adenoviral genomic DNA extracted from 293FT cells expressing Cas9:gRNA (173, 174, or 175) and infected with ADV-EGFP (P1); bottom: SURVEYOR assay of adenoviral genomic DNA extracted from AD293 cells infected with P1 progeny virus (P2). (D) Schematic of Cas9, which is guided by gRNA-175, hybridizing with the *EGFP* coding sequence. The protospacer-adjacent motif (PAM) sequences are highlighted in orange. (E) Sequences of indel mutations identified in 20 clones. Red dashes, deletions; red bases, insertions.

We chose the commonly used ADV to study whether DNA viruses could be repaired and mutated after nuclease cleavage. To exclude the effects of viral gene inactivation on viral replication, we selected the nonessential heterologous gene Enhanced Green Fluorescent Protein (*EGFP*) carried by a recombinant adenoviral vector (ADV-EGFP) as the target gene. Three guide RNAs (gRNA-173, gRNA-174, and gRNA-175) were designed to target the coding sequence of the *EGFP* gene (Figure 1A). When the vector DNA carrying the CRISPR-Cas9 system (pcw173, pcw174, and pcw175) was transfected into 293FT cells (Figure 1B), Cas9 protein expression was detected (Figure S1A). At 24 hours post-transfection, the cells were infected with ADV-EGFP at a multiplicity of infection (m.o.i.) of 1 PFU/cell, and the progeny viral genomes (P1) were harvested when cytopathic effects (CPEs) occurred. A SURVEYOR nuclease assay was then performed to detect site-specific mutations. All three gRNAs could induce site-specific mutations in the adenoviral genome (Figure 1C); gRNA-175 was the most efficient (47.4%), followed by gRNA-174 (37.3%), whereas gRNA-173 was the least efficient (32.7%). We further analyzed gRNA-175 by cloning the PCR products from the gRNA-175-cleaved viral genome into T-vectors for sequencing to determine the types of mutations that were present (Figure 1D). Nine of the twenty clones were wild type (containing no mutations), and eleven clones had indels in the region complementary to the gRNA-175. Five of these eleven clones had a GCG sequence missing, and three had a G missing, which are both believed to be dominant mutations. Additionally, each other type of mutation was found in one or two samples (Figure 1E). We next examined whether such mutations could be passed down to progeny viruses by infecting cells with the P1 virus and extracting the progeny viral genomes (P2) when CPEs occurred. A mutated genome was observed among the infectious P2 viruses that were analyzed using the SURVEYOR assay (Figure 1C). Our data indicate that DNA viral genomes can be repaired through NHEJ after site-specific cleavage by the CRISPR-Cas9 system and that the mutant genomes can be effectively transferred to progeny viruses. Moreover, the more active Cas9 resulted in more mutations in the progeny virus contained.

**Figure 2:**
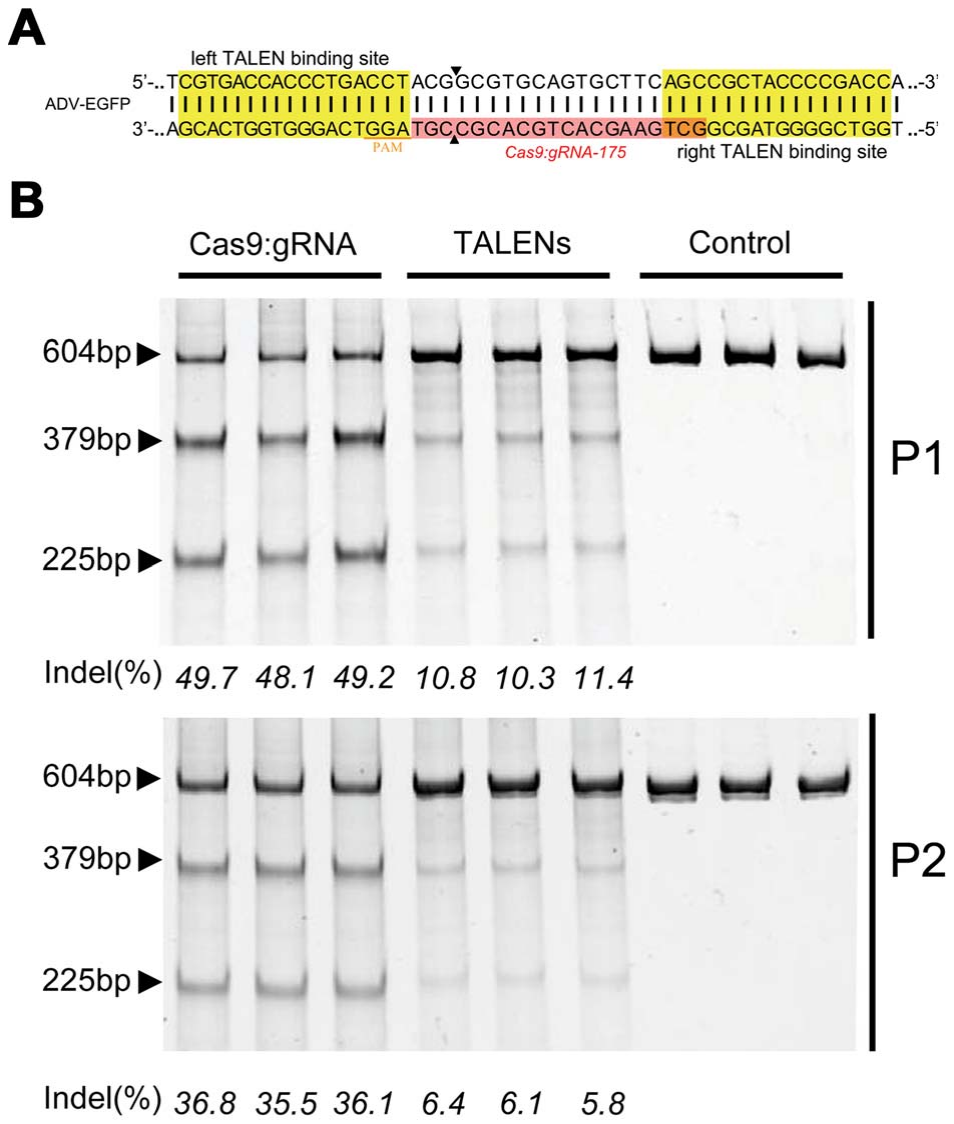
Comparison of the cleavage efficiencies of Cas9 and TALENs on viral genomes. (A) Schematic showing the design of TALENs targeting the *EGFP* gene in the ADV-EGFP genomes. (B) SURVEYOR gel comparing the efficiency of TALEN and Cas9 (n = 3).

To evaluate the advantages of the CRISPR-Cas9 system, we compared the efficiency of gene editing between CRISPR-Cas9 and a pair of TALENs targeting the same adenoviral locus (Figure 2A). The CRISPR-Cas9 system performed gene editing at a higher efficiency than the TALENs (Figure 2B).

Furthermore, the ratio of mutant to total viral genomes decreased dramatically from P1 to P2 during infectious viral particle packaging. Therefore, Cas9-mediated indel-mutated genomes were less likely to form infectious viral particles than wild-type viral genomes.

**Figure 3:**
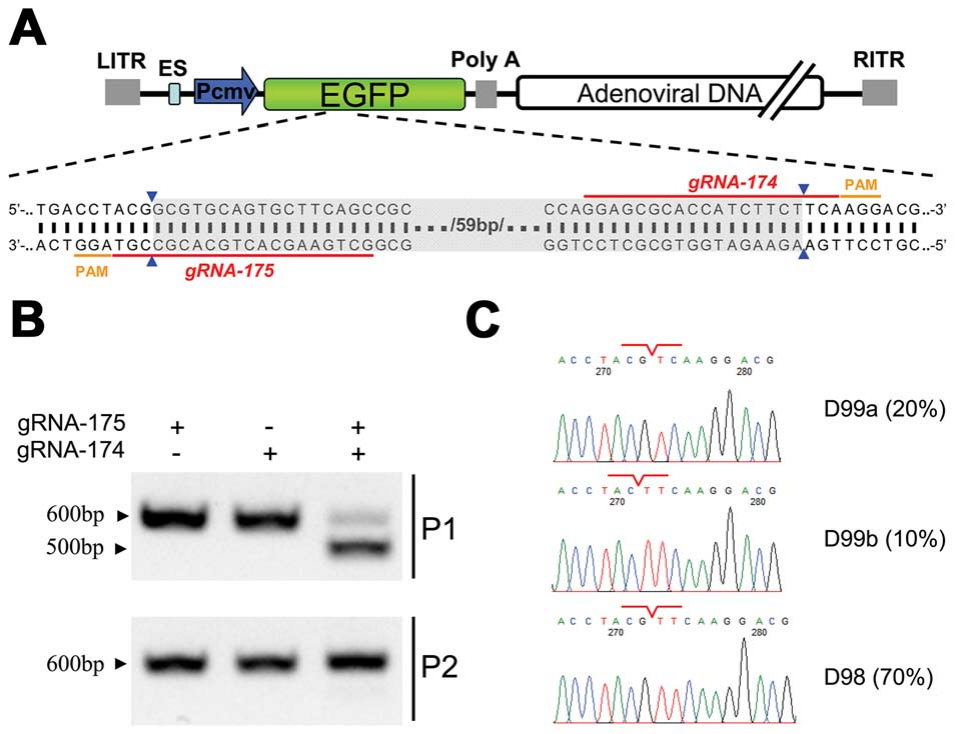
Cas9-targeted double genomic loci in recombinant adenoviral genomes. (A) Schematic of the *EGFP* gene in a recombinant adenoviral genome with the target sites of gRNA-175 and gRNA-174 indicated by red lines and the corresponding PAM in orange. The expected deletion is highlighted in grey. (B) Electrophoresis of the PCR products, including gRNA-174 and gRNA-175 target sites amplified from recombinant adenoviral genomes. Top: adenoviral genome extracted from 293FT cells expressing Cas9:gRNA and infected with ADV-EGFP (P1). Bottom: adenoviral genome extracted from AD293 cells infected with P1 progeny virus (P2). (C) Example chromatogram showing sequences of double gRNA-mediated (175 and 174) genomic deletions. The junction sites are indicated by red lines. The incidence of each genotype in ten clones is listed in the right-most column.

A more significant example of this phenomenon was demonstrated by co-expressing the Cas9 protein with gRNA-174 and gRNA-175 in cells to induce simultaneous double site-specific cleavages within the viral genome (Figure 3A). When the viral genome was amplified by PCR, this double cleavage resulted in the formation of a DNA fragment that was approximately 100 bp shorter (Figure 3B) than the product of single site-specific cleavages by gRNA-174 or gRNA-175. Further sequencing confirmed that the viral genomes were repaired at the exact gRNA-174 and gRNA-175 cleavage sites (Figure 3C). However, few of the viral genomes that resulted from double site-specific cleavages could form infectious viral particles, and the mutations in these genomes were rarely passed to the viral progeny when progeny viruses were used to infect fresh cells.

Because EGFP is a nonessential protein in the adenoviral replication cycle, our preliminary results revealed that viral genomes could be repaired by cellular DNA repair systems after Cas9 cleavage. Moreover, indels were formed and could be transferred to the progeny virus.

Because of the off-target activity of Cas9, we aligned the gRNA-175 sequence with ADV-EGFP genome. Based on previous reports that non-specific cleavage of CRISPR-Cas9 is sensitive to the position and number of mismatches, two methods were used to find homologous sequences. One approach was to find the longest concatenated sequence that matched the protospacer-adjacent motif (PAM)-proximal region. Until the PAM-upstream gRNA sequence was decreased to 7 nt, only one homologous sequence other than the target sequence was found. The number of mismatches between gRNA-175 and this sequence was ten (OTC175-A1) (Table S3). The other method was searched for a homologous sequence with the closest match to the gRNA-175 base pairs, for which the most similar homologous sequence contained five mismatched base pairs and included 2 gaps. However, this sequence contained no PAM motif that *Streptococcus pyogenes* Cas9 could recognize (OTC175-B1). Among those containing the PAM homologous sequence, the fewest number of mismatched base pairs was seven (OTC175-B4) (Table S4). Using RGN-treated (gRNA-175) P1 ADV-EGFP progeny viral genomes as the object, deep sequencing was performed on these three highly homologous regions, and no off-target mutations were detected (Table 1). Furthermore, an RGN (gRNA-175)-induced ADV-EGFP mutant without green fluorescence was purified (ADV-EGFPdG), and whole-genome sequencing revealed only one guanine deletion in the gRNA-175 target site compared with ADV-EGFP genome (Text S2). These results suggest that the CRISPR-Cas9 system avoids off-target activity on adenoviral genomes.

**Table 1:** On- and off-target mutations in viral genomes induced by Cas9:gRNA-175 in the ADV-EGFP progeny virus.

Site name	No. of mismatches	Sequence (5′-3′) GCTGAAGCACTGCACGCCGTNRG	Indel mutation frequency (%)±SEM	Locus
Target-175	0	GCTGAAGCACTGCACGCCGTAGG	42.8±3.4	(−)1196-1174
OTC175-A1	10	G**ACCGT**G**TCTG**G**A**ACGCCGTTGG	N.D.	(+)2228-2250
OTC175-B1	5	GCTGAAGCA **- -**GC**G**CG**T**CGTAG**A**	N.D.	(−)11086-11066
OTC175-B4	7	GCT**C**A**CC**CAC**CC**CA**T**GCC**A**TGGG	N.D.	(−)5185-5163

OTC indicates an off-target candidate (with the number of sites as shown in Supplementary Tables S3, S4). Mismatches in the target sequence (20-nt gRNA175 hybrid region and 3-nt PAM sequence) are bolded and underlined. The mean indel mutation frequencies were determined using deep sequencing (N = 3). N.D., none detected. The sequence locus indicates the position on ADV-EGFP genome. (−), negative strand; (+), positive strand.

### Factors that determine viral genome editing efficiency

Because of the convenience of downstream recombinant virus isolation, factors affecting viral genome editing were further evaluated.

**Figure 4:**
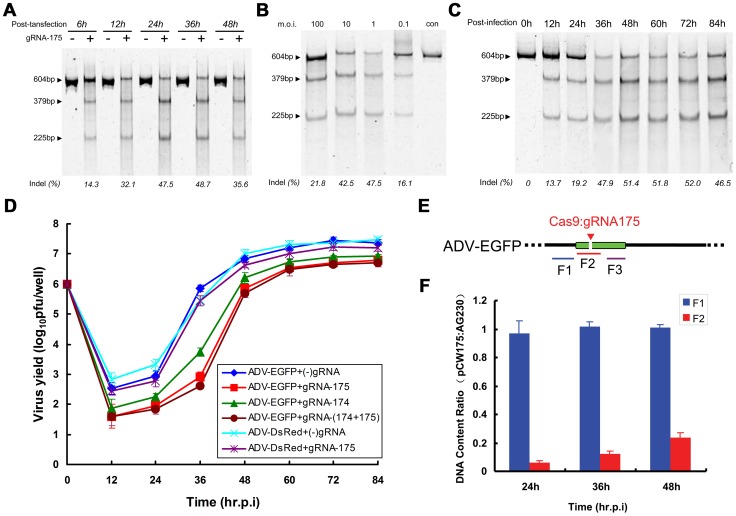
Factors affecting the efficiency of Cas9-mediated viral genome indels during adenovirus infection. SURVEYOR assay of adenoviral genomes extracted from 293FT cells expressing Cas9:gRNA-175 infected with ADV-EGFP at various time points after transfection (A), using various multiplicities of infection (B), and at different cell harvest time points after viral infection (C). (D) Viral growth curve comparison for recombinant adenovirus-infected 293FT cells expressing the Cas9:gRNA complex or Cas9 protein alone. (E) Schematic showing the F1–F3 regions located in ADV-EGFP viral genomes quantified using qPCR. The Cas9:gRNA-175 target site is indicated. (F) The quantity of the F2 DNA region in infected cells was compared between Cas9:gRNA175-treated ADV-EGFP and the control (Cas9 only). F3 was used to normalize the viral genome content in the different samples, and F1 served as a control. N = 3; error bars show the means ± SEM.

We first examined the effect of post-transfection infection time on the efficiency of mutant formation when introducing the CRISPR-Cas9 system into cells. The mutation efficiency increased with the increase in Cas9-gRNA (pcw175) expression and reached its peak between 24 and 36 hours post-transfection (Figure 4A), which is the peak time for the expression of heterologous genes post-plasmid transfection and the time when Cas9 accumulates in the nucleus (Figure S1B). We next studied the effect of various viral m.o.i. on the efficiency of mutant formation (Figure 4B). Mutant viral genome formation was the most efficient at an m.o.i. of between 1 and 10, and the efficiency of Cas9 cleavage and NHEJ repair decreased at a viral m.o.i. of 100, likely due to excessive quantities of viral genomes entering the cells. Consequently, the viral genome may be completely cleaved and repaired at a low m.o.i. Surprisingly, at a viral m.o.i. of 0.1, the mutation efficiency declined, and at this relatively low m.o.i., no significant CPE was observed, even after a long incubation period post-CRISPR-Cas9 transfection (up to 6 days); this result indicated that at a low m.o.i., viral genomes cannot effectively replicate in cells transfected with the CRISPR-Cas9 system. In the few cells that were not transfected with CRISPR-Cas9 (approximately 5%), the wild-type virus replicated normally; therefore, the percentage of mutants decreased. If a sufficient m.o.i. was maintained, the proportion of mutant virus among viral progeny could be further improved after two rounds of Cas9 cleavage (Figure S2).

Finally, we evaluated the proportion of mutant progeny viral genomes among the total viral genomes at various harvest times. Viral genomes mutated by Cas9 cleavage and subsequent repair were detectable at 12 hours post-infection. As time progressed, the percentage of mutant progeny genomes reached a maximum and was maintained at 47%-52% of the total genomes at 36 hours (Figure 4C).

We also detected a change in viral titer during sampling. In control cells, the viral logarithmic growth phase occurred at 24–36 hours post-infection, although in RGN (gRNA-175)-treated cells, the logarithmic growth phase was delayed to 36–48 hours, and the peak titer was 4–5-times lower than the viral titer of control cells (Figure 4D). When the guide RNA was substituted with gRNA-174, the replication of ADV-EGFP was also inhibited. Moreover, the viral replication was further reduced when gRNA175 and gRNA174 were expressed. However, when the virus was substituted with ADV-DsRed harboring a red fluorescent protein from *Discosoma coral* (*DsRed*), specific gRNA-175-induced RGN did not interfere with the viral replication process (Figure 4D). These results suggest that RGN can specifically inhibit the replication of viral genomes carrying a sequence complementary to the gRNA.

To investigate the mechanism by which RGN inhibits viral replication, we examined the repair efficiency after ADV-EGFP was cleaved by Cas9:gRNA-175. This experiment quantitatively detected three sequence regions (F1–F3) in the ADV-EGFP genome (Figure 4E). The cleavage site that gRNA-175 recognizes is within the F2 region, and the complete, but not the cleaved, genome can be amplified to obtain the F2 PCR product. F1 and F3 lie up- and down-stream of the gRNA-175 complementary sequence, respectively, and F3 was used to normalize the quantities of F1 and F2 in the two samples. In RGN (gRNA-175)-expressing cells, the quantity of F1 was equal to that of the control, whereas F2 was significantly lower than that in the control (Figure 4F). These results suggest that, in RGN-expressing cells, most of the viral genomes are in a cleaved form. At 24 hours post-viral infection, only 6.4±1.1% of the viral genomes were intact. Considering that the percentage of indels was 19.2% among the total F2 genomes (Figure 4C), we predicted that at 24 hours post-infection, more than 90% of the viral genomes were cleaved, and between 1% and 7% of the viral genomes were repaired by the host DNA repair machinery. With an increase in viral replication, the proportion of the F2 fragment increased, and at 48 hours post-infection, the proportion of complete viral genomes among the total viral genomes within cells reached 23.8±3.0%.

Our results indicate that the cellular expression level of the RGN complex, viral m.o.i., and viral harvest time all affect the formation of mutant viral progeny. In addition, cleavage by the CRISPR-Cas9 system significantly inhibits viral replication.

### RGN enhances adenoviral genome homologous recombination in a packaging cell line

**Figure 5:**
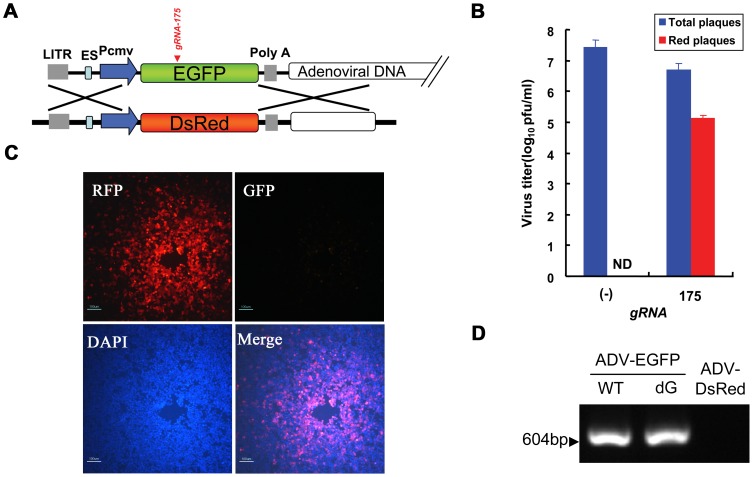
The substitution of genes on an adenoviral vector using Cas9:gRNA-induced HDR. (A) Strategy for Cas9:gRNA-induced HDR used to replace the *EGFP* gene in the adenoviral genome with the *DsRed* gene from donor DNA (pCW167). The Cas9:gRNA-175 target site is indicated in red. (B) Titration of recombinant adenovirus using a plaque assay. HDR was induced by the Cas9 protein or Cas9:gRNA-175. Blue bars represent the total number of macroscopically visible plaques. Red bars represent the number of red fluorescent plaques that were counted using a fluorescence microscope. N>5; error bars indicate the means ± SEM. ND, not detected. (C) An isolated ADV-DsRed plaque that was observed using different filters on a fluorescence microscope, and a merged image of a DAPI-stained nucleus and red fluorescent protein (RFP). Scale bar, 100 µm. (D) PCR amplification using oligo 1 and oligo 2 and adenoviral genomes extracted from a red fluorescent plaque to verify the lack of wild-type ADV-EGFP or indel mutant (such as ADV-EGFPdG) contamination.

Homologous recombination is induced to repair DNA in the presence of donor DNA, DSBs caused by wild-type Cas9 cleavage, and nicks caused by Cas9n nickase mutant cleavage [8]. Therefore, this endogenous mechanism can be exploited to introduce heterologous genes that are carried on a viral vector. To analyze the efficiency and feasibility of this approach, we introduced donor DNA encoding *DsRed* (pcw167) while transfecting the CRISPR-Cas9 system (pcw175) into cells (Figure 5A). The titer of the viral progeny obtained from cells infected with recombinant adenovirus was detected using a 10-fold serial dilution plaque assay. Viral titers decreased by 0.7–1.1 orders of magnitude (approximately 4–10-fold) in the presence of gRNA compared with a control that contained wild-type Cas9 protein alone. Fluorescence microscopy demonstrated no expression of red fluorescent protein in fresh cells infected with control progeny virus, which suggests that the efficiency of homologous recombination was lower than 10^−7.4^. However, red fluorescent protein expression was observed at multiple dilution factors of fresh cells that were infected with progeny virus and were obtained from the RGN system, and the expression of red fluorescent protein was still observed in progeny virus, even at dilutions of 10^5^. The efficiency of homologous recombination increased to 2.6±0.57% (Figure 5B), which was a significant increase compared with naturally occurring homologous recombination. Individual red plaques could be observed and isolated after 4–6 days of incubation due to the high recombination efficiency. A plaque was selected and examined after viral amplification by PCR and sequencing to further confirm that the progeny viruses were single ADV-DsRed (Figure 5C, 5D). Moreover, whole-genome sequencing of ADV-DsRed confirmed that no other mutations were induced (Text S2).

**Figure 6:**
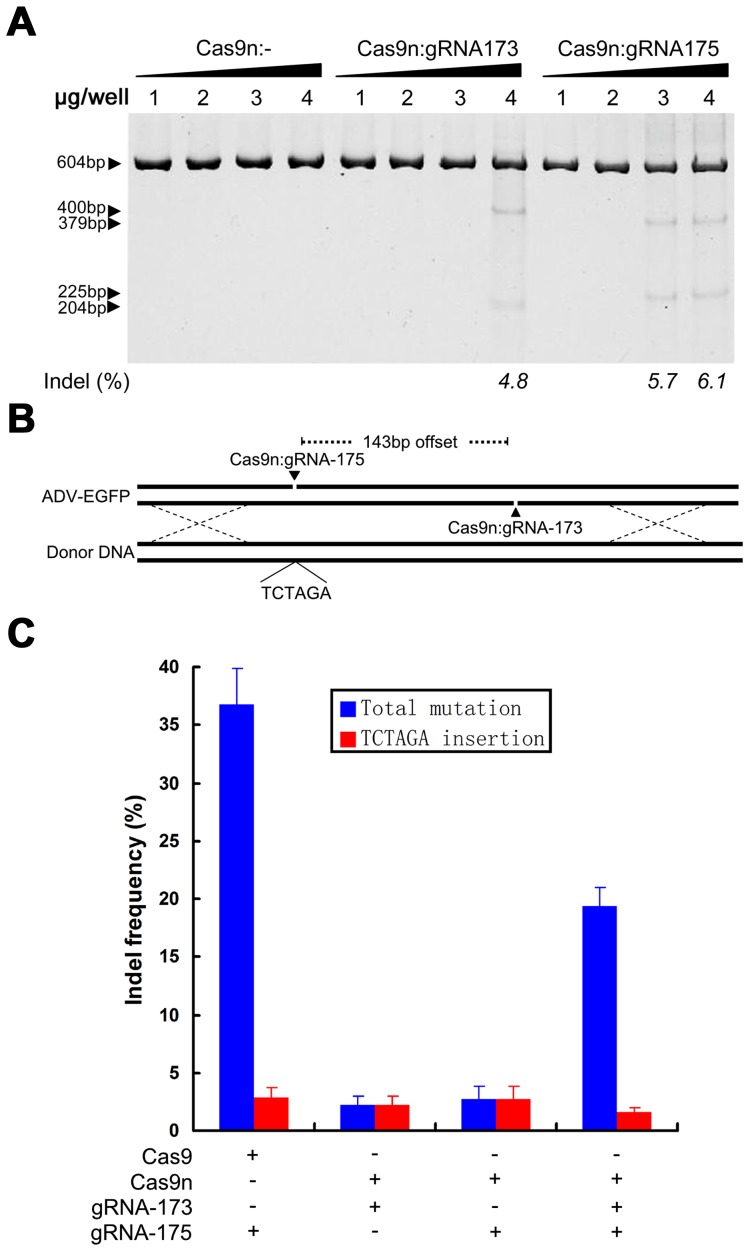
Specific control of the type of mutation using Cas9 nickase. (A) SURVEYOR assay of adenoviral genomes extracted from 293FT cells transfected with various amounts of a bicistronic vector co-expressing Cas9n and gRNA-173 or gRNA-175 and infected with ADV-EGFP. (B) Schematic showing the insertion of an XbaI restriction site into an ADV-EGFP genome via donor DNA and Cas9n. (C) Single nicking-mediated HDR specifically controls the types of mutation in progeny viruses (P2). Mutation types and HDR frequencies were determined using deep sequencing. N = 3; error bars show the means ± SEM.

To specifically control for the types of Cas9-mediated mutations, the D10A nickase mutant of Cas9 was used to site-specifically edit the adenoviral genome [28]. High-concentration Cas9n could still introduce indels into the adenoviral genome (Figure 6A) and is different from that used to site-specifically edit cellular genomes [28]. Deep sequencing results indicated that by controlling the concentration of Cas9n that was introduced into cells in the presence of donor DNA (pcw270) (Figure 6B), an appropriate amount of Cas9n:gRNA (1.5 µg pcw180 or pcw178) could induce homologous recombination with donor DNA in viral genomes. The efficiency of homologous recombination by Cas9n was slightly lower than that of the wild-type Cas9 protein but could prevent the formation of other indels. By choosing a pair of Cas9n:gRNAs, even with an offset of up to 140 bp, several other types of indels could be introduced into the viral genome (Figure 6C). These results indicate that unlike cells, a single-site nickase combined with donor DNA can generate more precise viral mutants for relatively small viral genomes.

### One-step generation and single-round isolation of recombinant HSV by RGN-mediated viral genome editing

We performed gene knockouts and rapid reporter gene knock-ins in wild-type HSV1 to further establish the CRISPR-Cas9 system as a universal tool for large mutant viral genome construction. For convenient mutant efficiency validation, the *UL23* gene encoding thymidine kinase (TK), which has enzymatic activity, was chosen as the target for Cas9.

**Figure 7:**
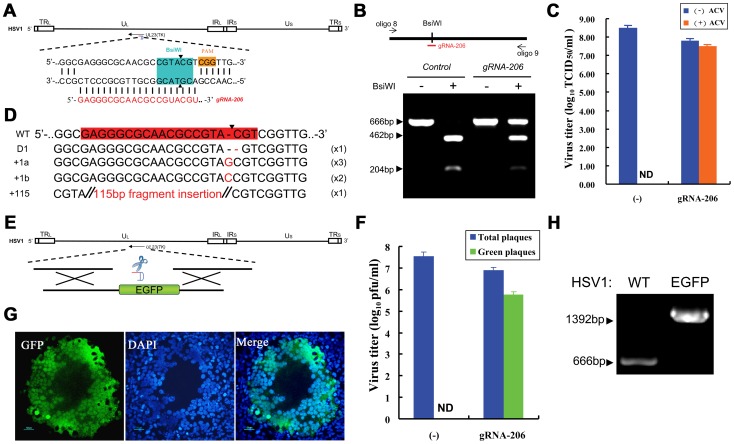
Herpes simplex viral genome editing targeted by the CRISPR-Cas system. (A) Schematic of the HSV1 genome and the gRNA-206 target site in the *UL23* gene. PAM sequences are highlighted in orange. The BsiWI restriction site used for RFLP is highlighted in aqua green. (B) The fragment amplified from an HSV1 genome using oligo 8 and oligo 9 was used in the RFLP assay. Co-expression of Cas9 and gRNA-206 in HSV1-infected cells leads to indel mutations in the *TK* gene of the HSV1 genome, which destroys the BsiWI restriction site. Arrows indicate fragments generated by BsiWI digestion. (C) Titration of HSV1 using an endpoint dilution assay in the presence or absence of 100 µg/ml ACV. The titers of total HSV1 progeny are represented by a blue bar. The titers of ACV-resistant HSV1 progeny virus are represented by an orange bar. N = 3; error bars show the means ± SEM. ND, not detected. (D) Sequences of indel mutations identified from eight ACV-resistant HSV1 strains. Red dashes, deletions; red bases, insertions. The incidence of each genotype is listed in the right-most column. (E) Strategy of Cas9:gRNA-induced HDR used to insert the *EGFP* gene into the HSV1 genome using donor DNA. (F) Titration of recombinant HSV1 using a plaque assay. HDR was induced by the Cas9 protein or Cas9:gRNA206. Blue bars represent the total number of macroscopically visible plaques; and green bars represent the number of green fluorescent plaques that were counted using a fluorescence microscope. N>5; error bars show the means ± SEM. ND, not detected. (G) An isolated green HSV1 plaque that was observed using a fluorescence microscope and merged with an image of DAPI staining. Scale bar, 50 µm. (H) PCR amplification of HSV1 genomes extracted from a green fluorescent plaque using oligo 8 and oligo 9 to verify the lack of wild-type HSV1 contamination.

We selected gRNA-206 on *UL23* as the target sequence, and the cleavage site that was induced by gRNA-206 was within the cleavage site of the restriction enzyme BsiWI (Figure 7A). In the absence of RGN (pcw206), the PCR product was completely digested by BsiWI; and two fragments, 462 bp and 204 bp, were produced. However, in the presence of RGN, more than 50% of the fragments within the HSV1 genome could not be cleaved (Figure 7B). These results suggest that the HSV1 genome was efficiently cleaved by Cas9 and that the BsiWI restriction cleavage site was destroyed through cellular NHEJ, which repaired the indels. We then used endpoint dilution assays to measure the titers of the progeny viruses collected at different time points. The viral growth curves showed inhibition of the HSV1 replication by Cas9:gRNA-206 (Figure S3). At 36 hours post infection, the titer for control viral progeny lacking RGN was 3.2×10^8^ TCID_50_/ml, and the titer decreased to 6.31×10^7^ TCID_50_/ml for the viral progeny with RGN. A known treatment for herpes infection is acyclovir (ACV), a drug that targets TK. Drug-resistant, but not wild-type, viral strains can survive in the presence of 100 µg/ml ACV during titration. No ACV-resistant viruses were isolated from the viral progeny produced by the control cells; however, in the presence of RGN, ACV-resistant viral progeny reached titers of 3.16×10^7^ TCID_50_/ml (Figure 7C), which was 50.1% of the total virus. PCR and sequencing were performed on virus extracted from eight wells of cells with CPE at the highest dilutions (10^−6^ and 10^−7^) (Figure S4A). Sequencing indicated that seven of the eight wells contained single viral mutant (Figure 7D), all of which included indels at the gRNA-206 site within the *UL23* sequence. Of these eight mutants, one was missing a cytosine (D1), three harbored a guanine insertion (+1a), two contained a cytosine insertion (+1b), one had an insertion of a 115-bp sequence (+115, see Figure S4B for sequence), and one was a mixture of two mutant viruses (Figure S4C). All ACV-resistant progeny viruses underwent site-directed mutagenesis within the *UL23* gene.

The homologous sequence of gRNA-206 was aligned with the HSV1 viral genome, and the most similar PAM-proximal sequence in the genome contained nine concatenated identical base pairs and nine mismatches (OTC206-A1) (Table S5). Moreover, the most similar homologous sequence to gRNA-206 in the HSV1 genome contained five mismatches and included a gap or six mismatches within the PAM sequence (Table S6). Deep sequencing was performed on these regions after RGN cleavage, and no mutations were detected (Table 2). These results further demonstrate that, when using the CRISPR-Cas9 system to edit viral genomes, off-target effects can be avoided.

**Table 2:** On- and off-target mutations in viral genomes induced by Cas9:gRNA-206 in the HSV1 progeny virus.

Site name	No. of mismatches	Sequence (5′-3′) GAGGGCGCAACGCCGTACGTNRG	Indel mutation frequency (%)±SEM	Locus
Target-206	0	GAGGGCGCAACGCCGTACGTCGG	49.4±4.7	(+)47709-47731
OTC206-A1	9	**AG**G**A**G**GTACGT**GCCGTACGTGGG	N.D.	(+)43002-43024
OTC206-B1	5	**AG**GGGCGC**C**ACG**G**CG**-**ACGTCGG	N.D.	(+)61902-61923
OTC206-B2	6	GA**CAA**CGC**G**ACGCCGT**T**CG**G**CGG	N.D.	(−)58600-58578

OTC indicates an off-target candidate (with the number of sites as shown in Supplementary Tables S5, S6). Mismatches in the target sequence (20-nt gRNA206 hybrid region and 3-nt PAM sequence) are bolded and underlined. Mean indel mutation frequencies were determined using deep sequencing (N = 3). N.D., none detected. The sequence locus indicates the position on HSV1 genome (GenBank: NC_001806.1). (−), negative strand; (+), positive strand.

To construct HSV1 carrying an *EGFP* reporter gene, we introduced homologous donor DNA (pcw209) and used RGN to cleave the viral genome (Figure 7E). The progeny virus was assessed using a plaque assay and fluorescence microscopy. The efficiency of homologous recombination was less than 1.45×10^−8^ in control cells without RGN. In cells treated with RGN and donor DNA, (5.8±1.7)×10^5^ pfu/ml green plaques were obtained among (6.9±1.8)×10^6^ pfu/ml total plaques (Figure 7F), and the efficiency of homologous recombination increased to 8.41%. A single HSV1-EGFP viral plaque was isolated from a 10^−5^ dilution and verified by PCR as pure virus with no wild-type HSV1 contamination (Figure 7G, 7H). Therefore, the purified virus could be used directly for further amplification and incubation.

## Discussion

To the best of our knowledge, the use of the CRISPR-Cas system for editing a non-integrating viral genome has not been previously reported. HSV1 was used to exemplify the current genome editing approach, which requires amplification and purification to obtain viral genomic DNA. The genomic DNA is then co-transfected with homologous DNA sequences into cells followed by two-step counter-selection and multiple plaque isolation [30] or by inserting the viral genome into a BAC system and performing several *in vitro* molecular biological procedures [31]. Both processes require several weeks and are extremely laborious and time-consuming. However, in our study, by simply transfecting the CRISPR-Cas system into cells, infecting with virus, and performing an ordinary progeny virus isolation procedure, a purified viral mutant with a specific gene deletion, insertion, or sequence substitution can be obtained within a short period. Compared with well-known, site-specific genome editing technologies, such as ZFNs and TALENs, the new CRISPR-Cas approach is more convenient and efficient [10], [12].

Because the sequence specificity of *Streptococcus pyogenes* Cas9 tolerates 4 mismatches between the gRNA and complementary sequence, non-specific cleavage may occur in the cellular genome [12], [24], [25], [26], [27]. Sequence alignment indicated that 76 gRNA-175 off-target sites are present in the human genome, in which 19 are located in the exons of protein-coding genes (Table S7). The highest homology contains only 2 mismatches. At this site, the indel mutation frequency that is induced by Cas9:gRNA-175 was found to be 2.27±0.18% using deep sequencing. The off-target sites for gRNA-174, gRNA-173, and gRNA-206 that are present in the human genome are 123, 34, and 8, respectively. Because progeny viruses are able to infect new cells after cell lysis, the effect of host-cell mutations on progeny viral replication is limited. By contrast, no off-target sites containing fewer than 5 mismatches for those gRNAs exist in the ADV or HSV genomes. Therefore, for large viral genomes sized 40–1000 kb, non-specific cleavage should occur only rarely (Tables 1, 2, S3, S4, S5, S6). The guide RNAs that were used in the present study can specifically recognize a single target in the viral genome. However, it is noteworthy that, due to the compact structure of the viral genome, gene overlapping occurs; therefore, multiple genes might sometimes be affected by the single cleavage of a viral genome.

The CRISPR-Cas9 system accurately controls the site of mutation and is a highly efficient strategy for the construction of large recombinant viral genomes. Based on our experiments, high proportions of site-specific viral mutants and recombinant viruses were obtained using CRISPR-Cas9 through at least two mechanisms. First, the high cleavage efficiency of the system can significantly inhibit the replication of the wild-type virus, and second, breakage at the cleavage site can efficiently induce NHEJ or HDR.

CRISPR-Cas9-induced cellular genome repair efficiency is not a major concern for eukaryotic cells, but for viruses, it could be an issue. Our study demonstrates that large quantities of viral genomes cannot be efficiently repaired after cleavage, which is likely the major reason for the inhibition of viral replication. Because viral genomes are much smaller than cellular genomes, which are restricted to the nucleus, once the relatively dissociated viral genome is cleaved by a nuclease, gene breakage and separation may be more likely to occur. As a result, even high concentrations of Cas9n nickase in complex with gRNA can induce indels. Alternatively, due to the presence of hundreds of viral genomes within a single infected cell, the limited efficiency of the DNA repair systems within cells may not be able to repair the fragmented viral genomes in time. Therefore, only some cleaved viral genomes are repaired during the early stages of infection. However, mutant viruses possess replication priority under the cleavage pressure of the CRISPR-Cas9 system. As viral replication ensues, the proportion of viral mutants gradually increases, eventually reaching a high percentage of the total amount of virus. During the process, the formation of a high proportion of viral mutants is affected by factors, including infection time after Cas9 transfection, m.o.i., and time of harvest of viral progeny.

High cleavage efficiency and low cellular repair efficiency may limit the use of the CRISPR-Cas9 system to perform multiple-site editing of viral genomes within a single replication cycle. Moreover, the mutant virus that is formed in P1 cannot be passed to the P2 virus in equal proportions, which illustrates the steric hindrance caused by the association of the Cas9 protein with specific DNA sequences [34], and the time that elapses during cleavage and repair may affect viral genome packaging. Nevertheless, due to the short replication period and high progeny productivity of the virus, several rounds of CRISPR-Cas9 selection pressure could gradually increase the proportion of recombinant virus among total virus. This strategy could thus meet most of the genome reconstruction requirements of various types of large DNA viruses.

The high capacity of large DNA viruses allows them to be more sophisticated gene vectors, but the complexity of DNA virus life cycles and the difficulty of attenuated vaccine selection make it challenging to develop vaccines against them. The highly effective, site-specific CRISPR-Cas9 genome editing system will promote more rapid development of large-genome viral vectors, attenuated vaccines against large DNA viruses, and an understanding of viral life cycles.

## Materials and Methods

### Cell culture and viruses

The human embryonic kidney (HEK) cell lines 293FT (Life Technologies, Carlsbad, CA, USA) and AD293 (Clontech, Palo Alto, CA, USA) and the African green monkey kidney cell line Vero (ATCC, Manassas, VA, USA) were maintained in Dulbecco's modified Eagle's Medium (DMEM; Corning, New York, USA) supplemented with 10% fetal bovine serum (FBS; Life Technologies), 100 U/ml penicillin, and 100 µg/ml streptomycin at 37°C with 5% CO_2_. The culture medium was changed to DMEM supplemented with 2% FBS after viral infection of 293FT, AD293, and Vero cells. ADV-EGFP [35] and HSV1 strain 8F [36] were cultured and titered on AD293 cells and Vero cells, respectively.

### Genomic DNA extraction and PCR amplification

Virus from infected 293FT cells was harvested at 48 hours post-infection, and viral genomic DNA was extracted using the Takara miniBEST Viral RNA/DNA Extraction Kit Ver.4.0 (Takara Bio Inc., Dalian, China). The genomic region surrounding the CRISPR target site of each gene was PCR amplified using Phusion high-fidelity DNA polymerase (New England Biolabs, Beverly, MA, USA) with primers oligo 1 and oligo 2 for ADV-EGFP and oligo 8 and oligo 9 for HSV1. The PCR products were purified using the Universal DNA Purification Kit (Tiangen, Beijing, China).

### SURVEYOR assay and sequencing analysis

Purified ADV PCR products (400 ng) amplified from the genomic DNA extraction were re-annealed and treated with SURVEYOR nuclease (Transgenomics, Omaha, NE, USA) according to the manufacturer's recommended protocol. The products were analyzed on 10% TBE polyacrylamide gels, which were stained with SYBR Gold DNA stain (Life Technologies) and imaged using a Bio-Rad Gel Doc gel imaging system (Richmond, CA, USA). Quantification was based on the relative band intensities, as described by Cong et al. (2013) [12]. The PCR products were ligated into the pMD20-T vector (Takara Bio Inc.) and submitted for sequencing using universal primers (BGI, Guangzhou, China).

### Restriction fragment length polymorphism (RFLP) analysis of gene modifications

Purified HSV1 DNA products from the genomic DNA extraction and PCR amplification were digested with BsiWI (New England Biolabs) for 16 hours at 55°C and analyzed on 0.5 µg/ml ethidium bromide-stained agarose gels (1%). Quantification was based on relative band intensities.

### Sequence alignment

To detect homologous sequences in the viral genome, gRNA-175 and gRNA-206 were aligned to the ADV-EGFP and HSV1 whole genomes, respectively, using two methods. gRNA sequences were sequentially deleted (from PAM-distal to PAM-proximal) and used to search for identical sequences in the viral genome using Vector NTI (Life Technologies). In addition, gRNA sequences were randomly aligned to viral genomic DNA using the EBI online sequence alignment tool (http://www.ebi.ac.uk/Tools/psa/lalign/nucleotide.html).

### Deep sequencing

Amplicon deep sequencing was performed on a 454/Roche GS Junior platform (Roche, 454 Life Sciences, Branford, CT, USA) according to the manufacturer's instructions. Each sample was amplified independently with different primers, including the 454 primer keys, and a different multiple identifier (MID) was used for each sample. The PCR products were resolved by 2% agarose gel electrophoresis and purified. The purified amplicons were quantified using the QuantiFluor dsDNA System (Promega, Madison, WI, USA), diluted, and subjected to emulsion PCR (emPCR). The enriched DNA beads were loaded onto a picotiter plate, and pyrosequencing was performed using titanium chemistry. Amplicon Variant Analyzer version 2.7 was used for the analysis.

### Quantitative PCR (qPCR)

Quantitative PCR (qPCR) was performed to analyze the repair efficiency after Cas9 cleavage of the viral genome. Viral genomic DNA from infected cells was extracted as described above, and qPCR was performed on an ABI 7500 (Life Technologies) using SuperReal PreMix Plus (SYBR Green) (Tiangen, Beijing, China). Three sets of sequences for ADV-EGFP were amplified separately (F1–F3). F1 was amplified using primers oligo 3 and oligo 4, F2 was amplified using primers oligo 1 and oligo 5, and F3 was amplified using primers oligo 6 and oligo 7. Each experiment was performed in parallel, and triplicate samples were included in each reaction.

## Supporting Information

Figure S1 Cas9 protein expressed in 293FT cells. (A) Lysate from 293FT cells transfected with a Cas9 expression plasmid was analyzed using Western blotting with anti-Flag antibodies. (B) The location of exogenous Flag-NLS-Cas9-NLS protein expression in 293FT cells detected using IFA with anti-Flag antibodies at 24 and 48 hours post-transfection.
(TIF)

Figure S2 Re-infection of 293FT cells expressing Cas9:gRNA-175 with ADV-EGFP to increase the proportion of indel mutations. (A) Schematic drawing of the experimental design. (B) SURVEYOR assay of adenoviral genomes extracted from various viral passages, as indicated in panel A.
(TIF)

Figure S3 Viral growth curve comparison for HSV1-infected 293FT cells expressing the Cas9:gRNA-206 or Cas9 protein alone.
(TIF)

Figure S4 Genotyping of ACV-resistant HSV1 mutants induced by Cas9:gRNA-206-mediated NHEJ. (A) Isolation of an ACV-resistant HSV1 mutant using an endpoint dilution assay. ACV-resistant HSV1 mutants, which were infected with the highest viral dilution (10^−6^, 10^−7^), were obtained from eight single CPE wells. The ACV-resistant HSV1 was genotyped using PCR and DNA sequencing. The sequencing results for cells from the two HSV1 wells (+115 and two virus mixture) are shown in panels (B) and (C). The others are displayed in Figure 7D.
(TIF)

Table S1 DNA sequences of gRNAs and primers used for plasmid construction.
(DOC)

Table S2 Primers used for the SURVEYOR assay, sequencing, RFLP analysis, and qPCR.
(DOC)

Table S3 Homologous target sequences of gRNA-175 that match the PAM-proximal region in the ADV-EGFP genome.
(DOC)

Table S4 Homologous target sequences of gRNA-175 in the ADV-EGFP genome.
(DOC)

Table S5 Homologous sequences of gRNA206 that match the PAM-proximal region in the HSV1 genome.
(DOC)

Table S6 Homologous target sequences of gRNA-206 in the HSV1 genome.
(DOC)

Table S7 Homologous target sequences of gRNA-175 in human protein-coding genes.
(DOC)

Text S1 Extended Materials and Methods.
(DOC)

Text S2 Supplementary sequences.
(DOC)
