# Supplementary figures and images for: High-Efficiency Targeted Editing of Large Viral Genomes by RNA-Guided Nucleases
Source: PLoS Pathog. 2014 May 1;10(5):e1004090. doi: 10.1371/journal.ppat.1004090 (PMC4006927; doi:10.1371/journal.ppat.1004090)

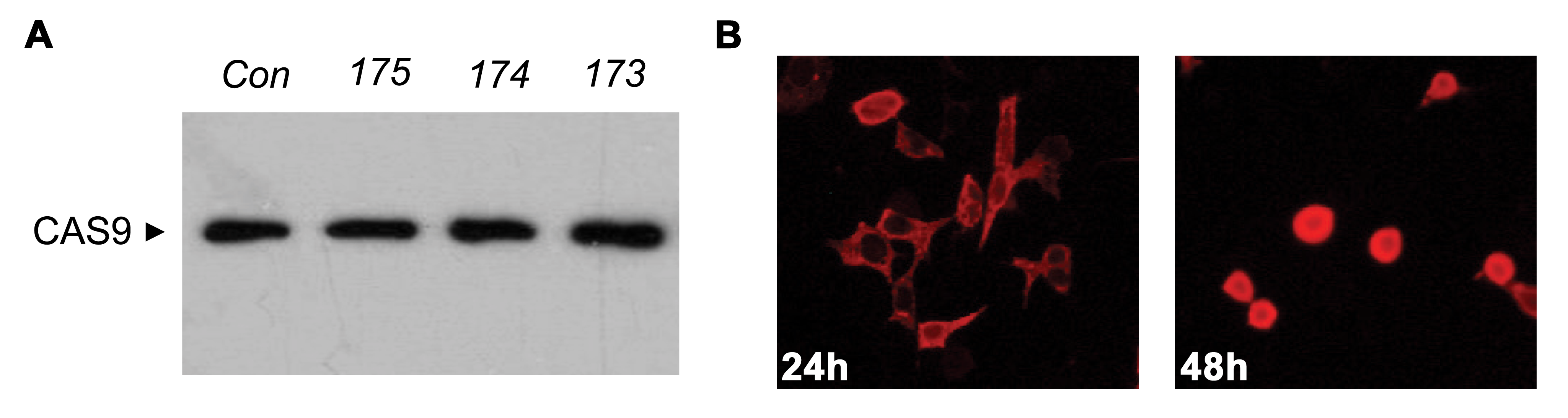

Supplement: Figure S1 — Cas9 protein expressed in 293FT cells. (A) Lysate from 293FT cells transfected with a Cas9 expression plasmid was analyzed using Western blotting with anti-Flag antibodies. (B) The location of exogenous Flag-NLS-Cas9-NLS protein expression in 293FT cells detected using IFA with anti-Flag antibodies at 24 and 48 hours post-transfection. (TIF) [file ppat.1004090.s001.tif]

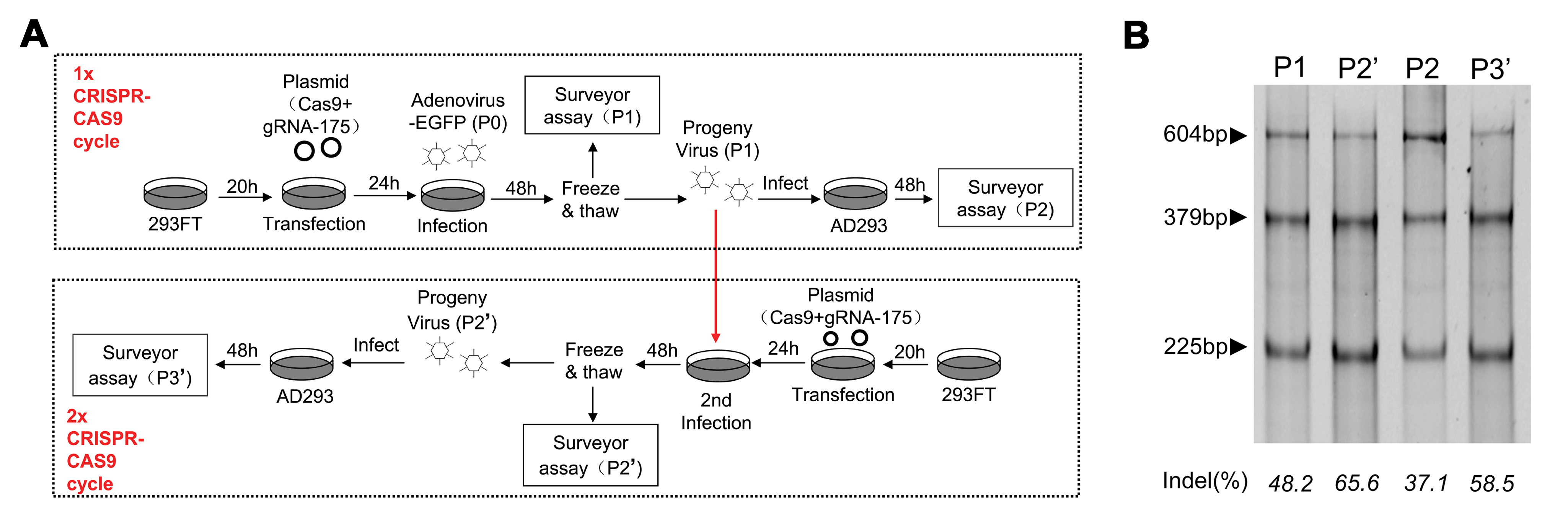

Supplement: Figure S2 — Re-infection of 293FT cells expressing Cas9:gRNA-175 with ADV-EGFP to increase the proportion of indel mutations. (A) Schematic drawing of the experimental design. (B) SURVEYOR assay of adenoviral genomes extracted from various viral passages, as indicated in panel A. (TIF) [file ppat.1004090.s002.tif]

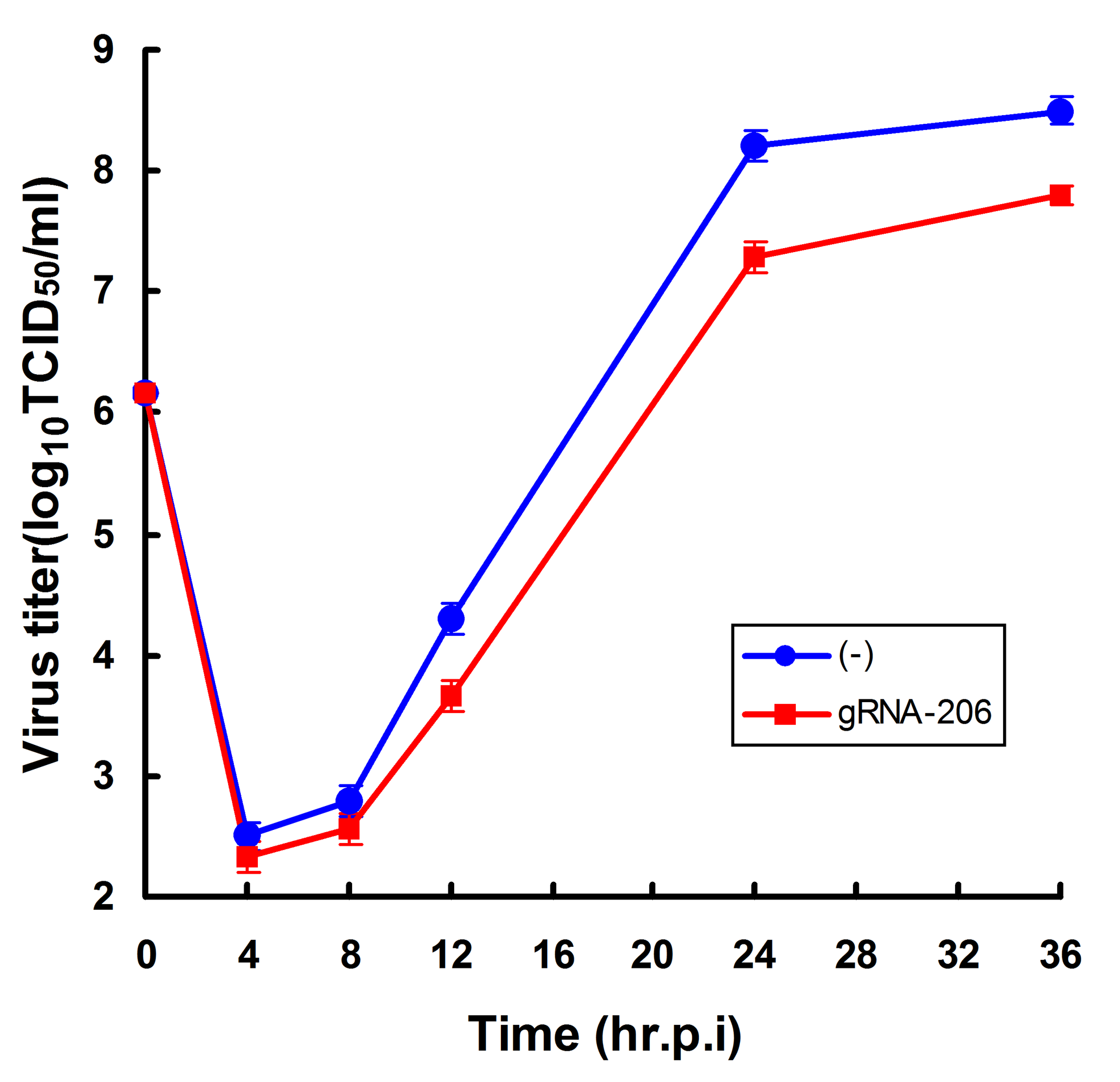

Supplement: Figure S3 — Viral growth curve comparison for HSV1-infected 293FT cells expressing the Cas9:gRNA-206 or Cas9 protein alone. (TIF) [file ppat.1004090.s003.tif]

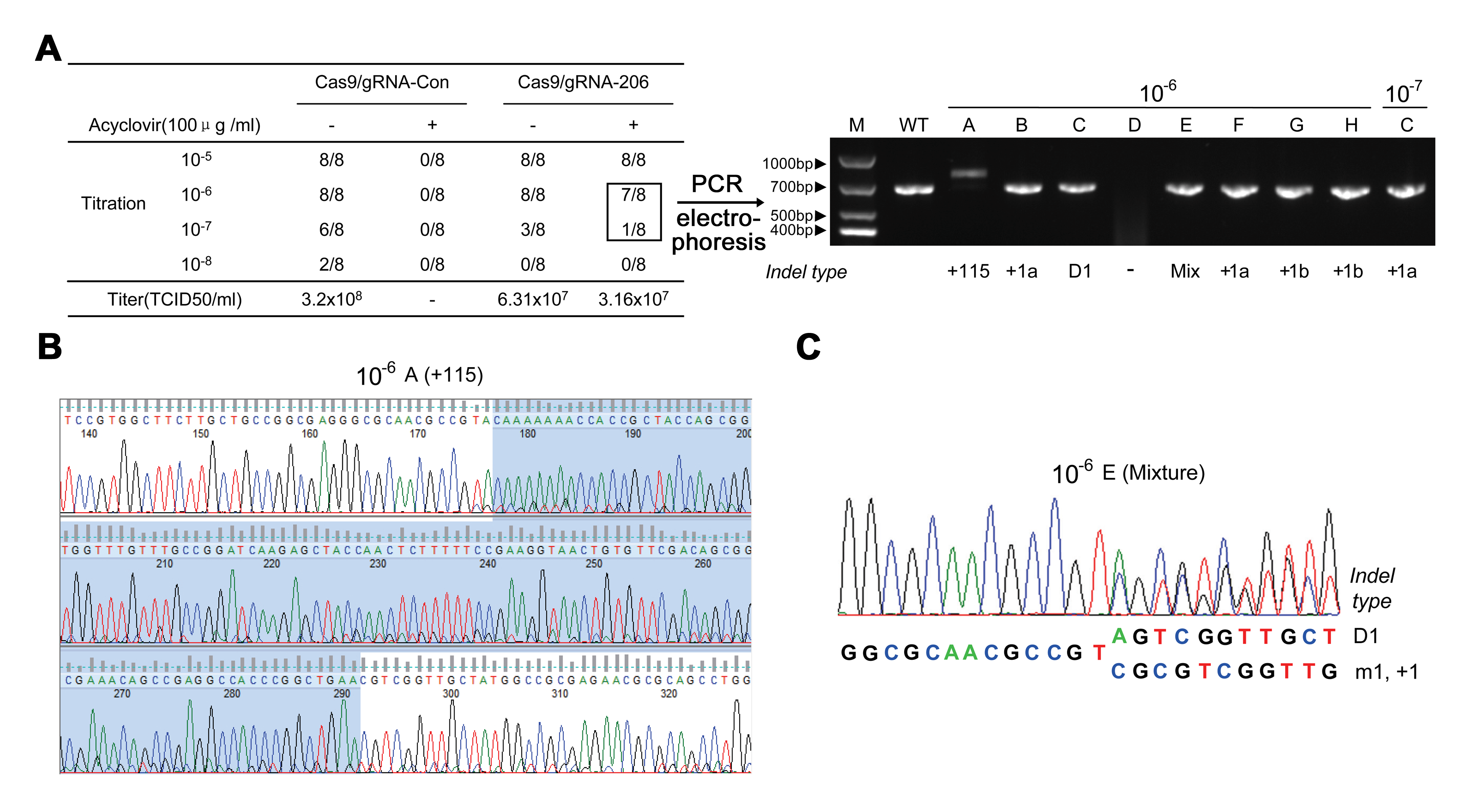

Supplement: Figure S4 — Genotyping of ACV-resistant HSV1 mutants induced by Cas9:gRNA-206-mediated NHEJ. (A) Isolation of an ACV-resistant HSV1 mutant using an endpoint dilution assay. ACV-resistant HSV1 mutants, which were infected with the highest viral dilution (10−6, 10−7), were obtained from eight single CPE wells. The ACV-resistant HSV1 was genotyped using PCR and DNA sequencing. The sequencing results for cells from the two HSV1 wells (+115 and two virus mixture) are shown in panels (B) and (C). The others are displayed in Figure 7D. (TIF) [file ppat.1004090.s004.tif]
