# Supplementary material for: High-Efficiency Targeted Editing of Large Viral Genomes by RNA-Guided Nucleases
Source: PLoS Pathog. 2014 May 1;10(5):e1004090. doi: 10.1371/journal.ppat.1004090 (PMC4006927; doi:10.1371/journal.ppat.1004090)
Supplement: Table S1 — DNA sequences of gRNAs and primers used for plasmid construction. (DOC) [file ppat.1004090.s005.doc]

Table S1. DNA sequences of gRNAs and primers used for plasmid construction

| Primer name | Assay | Genomic target | Primer sequence (5' to 3') |
| --- | --- | --- | --- |
| gRNA-173 F | pcw173 construction | EGFP | CACCGTGAACCGCATCGAGCTGAA |
| gRNA-173 R | pcw173 construction | EGFP | AAACTTCAGCTCGATGCGGTTCAC |
| gRNA-174 F | pcw174 construction | EGFP | CACCGGAGCGCACCATCTTCTTCA |
| gRNA-174 R | pcw174 construction | EGFP | AAACTGAAGAAGATGGTGCGCTCC |
| gRNA-175 F | pcw175 construction | EGFP | CACCGCTGAAGCACTGCACGCCGT |
| gRNA-175 R | pcw175 construction | EGFP | AAACACGGCGTGCAGTGCTTCAGC |
| gRNA-206 F | pcw206 construction | TK | CACCGAGGGCGCAACGCCGTACGT |
| gRNA-206 R | pcw206 construction | TK | AAACACGTACGGCGTTGCGCCCTC |
| w042 | pcw021 construction | TK | AATAAGCTTGGCGCCGCACCTCTTCGGCC |
| w043 | pcw021 construction | TK | CGGAATTCCCGCGGGTTCCTTCCGGTATTG |
| w286 | pcw209 construction | EGFP | TACCGTACGATGGTGAGCAAGGGCG |
| w287 | pcw209 construction | EGFP | TACCGTACGTTACTTGTACAGCTCGTCCATG |
| w340 | pcw270 construction | EGFP | GGTCTATATAAGCAGAGCTGGTTTAG |
| w341 | pcw270 construction | EGFP | CGCTCTAGACGTAGGTCAGGGTGGTC |
| w342 | pcw270 construction | EGFP | ACGTCTAGAGCGTGCAGTGCTTCAG |
| w343 | pcw270 construction | EGFP | GTGGTATGGCTGATTATGATCAG |

EGFP: Enhanced Green Fluorescent Protein; TK: Thymidine Kinase.
