# Supplementary material for: High-Efficiency Targeted Editing of Large Viral Genomes by RNA-Guided Nucleases
Source: PLoS Pathog. 2014 May 1;10(5):e1004090. doi: 10.1371/journal.ppat.1004090 (PMC4006927; doi:10.1371/journal.ppat.1004090)
Supplement: Table S2 — Primers used for the SURVEYOR assay, sequencing, RFLP analysis, and qPCR. (DOC) [file ppat.1004090.s006.doc]

Table S2. Primers used for the SURVEYOR assay, sequencing, RFLP analysis, and qPCR.

| Primer name | Assay | Genomic target | Primer sequence (5' to 3') |
| --- | --- | --- | --- |
| oligo 1 | SURVEYOR assay, sequencing, and F2 qPCR | EGFP | GGATCCACCGGCCGGTCG |
| oligo 2 | SURVEYOR assay and sequencing | EGFP | ACGGGGCCGTCGCCGATGG |
| oligo 3 | F1 qPCR | CMV promoter | GGGACTTTCCTACTTGGCAG |
| oligo 4 | F1 qPCR | CMV promoter | CTTGCTCACCATGGTGGCGAC |
| oligo 5 | F2 qPCR | EGFP | GGCGGACTTGAAGAAGTCG |
| oligo 6 | F3 qPCR | EGFP | GTGAACCGCATCGAGCTGAA |
| oligo 7 | F3 qPCR | EGFP | TTACTTGTACAGCTCGTCCATG |
| oligo 8 | RFLP and sequencing | TK | AAGCCCCCAGCACCCGCCAGTAAGT |
| oligo 9 | RFLP and sequencing | TK | CACCAGGACGGGGCACAGGTACACTATCTTG |

EGFP: Enhanced Green Fluorescent Protein; CMV: Cytomegalovirus; TK: Thymidine Kinase; RFLP: Restriction Fragment Length Polymorphism.
