# Supplementary material for: High-Efficiency Targeted Editing of Large Viral Genomes by RNA-Guided Nucleases
Source: PLoS Pathog. 2014 May 1;10(5):e1004090. doi: 10.1371/journal.ppat.1004090 (PMC4006927; doi:10.1371/journal.ppat.1004090)
Supplement: Table S3 — Homologous target sequences of gRNA-175 that match the PAM-proximal region in the ADV-EGFP genome. (DOC) [file ppat.1004090.s007.doc]

Table S3. Homologous target sequences of gRNA-175 matching the PAM-proximal region in ADV-EGFP genomes.

| nt | Sequence ^#^ (5´-3´) | Frequencies* (Site name) |
| --- | --- | --- |
| 22 | GCTGAAGCACTGCACGCCGTNRG | 1 (T175) |
| 10 | NNNNNNNNNNNNCACGCCGTNRG | 1 (T175) |
| 9 | NNNNNNNNNNNNNACGCCGTNRG | 2 (T175, OTC175-A1) |
| 8 | NNNNNNNNNNNNNNCGCCGTNRG | 4 (T175, OTC175-A1, A2, A3) |

#: N: A/T/G/C, R: A/G.

*: Frequencies at which homologous sequences appear in the ADV-EGFP genome;

T175 sequence (located in ADV-EGFP): GCTGAAGCACTGCACGCCGTAGG (1196-1174);

OTC175-A1 sequence (located in ADV-EGFP): GACCGTGTCTGGAACGCCGTTGG (2228-2250).
