# Supplementary material for: High-Efficiency Targeted Editing of Large Viral Genomes by RNA-Guided Nucleases
Source: PLoS Pathog. 2014 May 1;10(5):e1004090. doi: 10.1371/journal.ppat.1004090 (PMC4006927; doi:10.1371/journal.ppat.1004090)
Supplement: Table S4 — Homologous target sequences of gRNA-175 in the ADV-EGFP genome. (DOC) [file ppat.1004090.s008.doc]

Table S4. Homologous target sequences of gRNA-175 in the ADV-EGFP genome

| Mismatch number | Site name | Sequence^#^ (5´-3´) | Location* | |
| --- | --- | --- | --- | --- |
|  |  | GCTGAAGCACTGCACGCCGTNRG | start | stop |
| 0 | T175 | GCTGAAGCACTGCACGCCGTAGG | 1196 | 1174 |
| 5 | OTC175-B1 | GCTGAAGCA **- -** GC**G**CG**T**CGTAG**A** | 11086 | 11066 |
| 6 | OTC175-B2 | GCTGAAGC**G**CTGC**CG**G**T**C**T**TCG**C** | 3874 | 3896 |
| 6 | OTC175-B3 | G**T**TGAAGCA(T)CTGCA(G)CGCC**AG**TG**C** | 22821 | 22845 |
| 7 | OTC175-B4 | GCTGA**T**GCAC**A**GC**CT**GC**AAA**GGG | 10534 | 10556 |
| 7 | OTC175-B5 | G**TG**GAAGC**T**CTG**GCG**GCCGTA**T**G | 21866 | 21844 |
| 8 | OTC175-B6 | **A**CTG**C**AG**AG**CT**T**CA**T**GC**T**G**C**GGG | 2710 | 2732 |
| 8 | OTC175-B7 | **A**CTG**C**AGCAC**A**GCAC**CA**C**AA**TA**T** | 29231 | 29253 |

^#^: N: A/T/G/C, R: A/G, -: gap.

*: In the ADV-EGFP genome.

OTC indicates an off-target candidate. Mismatches from the target sequence (20-nt gRNA175 hybrid region and 3-nt PAM sequence) are bolded and underlined.
