# Supplementary material for: High-Efficiency Targeted Editing of Large Viral Genomes by RNA-Guided Nucleases
Source: PLoS Pathog. 2014 May 1;10(5):e1004090. doi: 10.1371/journal.ppat.1004090 (PMC4006927; doi:10.1371/journal.ppat.1004090)
Supplement: Table S5 — Homologous sequences of gRNA206 that match the PAM-proximal region in the HSV1 genome. (DOC) [file ppat.1004090.s009.doc]

Table S5. Homologous target sequences of gRNA206 matching the PAM-proximal region in the HSV1 genome.

| nt | Sequence (5´-3´) | Frequencies* (Site name) |
| --- | --- | --- |
| 22 | GAGGGCGCAACGCCGTACGTNRG | 1 (T206） |
| 12 | NNNNNNNNNNCGCCGTACGTNRG | 1 (T206) |
| 11 | NNNNNNNNNNNGCCGTACGTNRG | 2 (T206,OTC206-A1) |
| 10 | NNNNNNNNNNNNCCGTACGTNRG | 2 (T206,OTC206-A1) |
| 9 | NNNNNNNNNNNNNCGTACGTNRG | 6 (T206,OTC206-A1,A2,A3,A4,A5) |

^#^: N: A/T/G/C, R: A/G.

*: Frequencies at which homologous sequences appear in the HSV1 genome (Genbank: NC_001806.1);

T206 sequence (located in HSV1): GAGGGCGCAACGCCGTACGTCGG (47709-47731);

OTC206-A1 sequence (located in HSV1): AGGAGGTACGTGCCGTACGTGGG (43002-43024).
