# Supplementary material for: High-Efficiency Targeted Editing of Large Viral Genomes by RNA-Guided Nucleases
Source: PLoS Pathog. 2014 May 1;10(5):e1004090. doi: 10.1371/journal.ppat.1004090 (PMC4006927; doi:10.1371/journal.ppat.1004090)
Supplement: Table S6 — Homologous target sequences of gRNA-206 in the HSV1 genome. (DOC) [file ppat.1004090.s010.doc]

Table S6. Homologous target sequences of gRNA-206 in the HSV1 genome

| Mismatch number | Site name | Sequence^#^(5´-3´) | Location* | |
| --- | --- | --- | --- | --- |
|  |  | GAGGGCGCAACGCCGTACGTNRG | start | stop |
| 0 | T206 | GAGGGCGCAACGCCGTACGTCGG | 47709 | 47731 |
| 5 | OTC206-B1 | **AG**GGGCGC**C**ACG**G**CG**-**ACGTCGG | 61902 | 61923 |
| 6 | OTC206-B2 | GA**CAA**CGC**G**ACGCCGT**T**CG**G**CGG | 58600 | 58578 |
| 6 | OTC206-B3 | G**C**GGGC**T**CAACGCCGT**G**C**TG**GG**C** | 101642 | 101620 |
| 6 | OTC206-B4 | **T**AGGGC**C**-AACGCCGT**C**CG**G**C**T**G | 18839 | 18860 |
| 8 | OTC206-B5 | **CG**GG**A**CGCAACGCCGT**GA**G**G**C**TC** | 102117 | 102139 |

^#^: N: A/T/G/C, R: A/G, -: gap.

*: In the HSV1 genome (Genbank: NC_001806.1).

OTC indicates an off-target candidate. Mismatches from the target sequence (20-nt gRNA206 hybrid region and 3-nt PAM sequence) are bolded and underlined.
