# Supplementary material for: High-Efficiency Targeted Editing of Large Viral Genomes by RNA-Guided Nucleases
Source: PLoS Pathog. 2014 May 1;10(5):e1004090. doi: 10.1371/journal.ppat.1004090 (PMC4006927; doi:10.1371/journal.ppat.1004090)
Supplement: Table S7 — Homologous target sequences of gRNA-175 in human protein-coding genes. (DOC) [file ppat.1004090.s011.doc]

Table S7. Homologous target sequences of gRNA-175 in human protein-coding genes.

^#^: N: A/T/G/C, R: A/G,

| Mismatch  number | Site name | Sequence^#^ (5´-3´) | Gene |
| --- | --- | --- | --- |
|  |  | GCTGAAGCACTGCACGCCGTNRG |  |
| 2 | OTC175-C1 | GCTG**T**AGCACT**C**CACGCCGTTGG | *LYPD3* |
| 3 | OTC175-C2 | **A**CTGAAGCACTGCA**G**GC**A**GTGAG | *TNIP2* |
| 4 | OTC175-C4 | GC**G**GA**C**GCA**T**TGCACGCCG**G**GAG | *LLPH* |
| 4 | OTC175-C5 | GC**G**G**C**AGCA**G**TGCA**G**GCCGTGAG | *ADCY4* |
| 4 | OTC175-C6 | GC**C**GAAG**GC**CTGCACG**A**CGTGAG | *ATRAID* |
| 4 | OTC175-C7 | GCTGA**GT**C**C**CTGCA**G**GCCGTGAG | *GPR17* |
| 4 | OTC175-C8 | GCTGAAG**TC**CTGCACGC**A**G**G**TGG | *POLR1A* |
| 4 | OTC175-C9 | GCTGA**G**GC**C**CTGCA**G**GCCG**G**GGG | *C9orf142* |
| 4 | OTC175-C10 | GCTGAAGC**G**C**AC**CA**G**GCCGTGGG | *CPT1C* |
| 4 | OTC175-C11 | **C**CTGAA**A**CACTGCA**G**GC**T**GTCAG | *CALD1* |
| 4 | OTC175-C12 | GCTG**T**AGCACTGCA**G**G**G**C**T**TGGG | *LYPD5* |
| 4 | OTC175-C13 | GCTG**G**AGCACT**C**CAC**CA**CGTTGG | *SLIT1* |
| 4 | OTC175-C14 | GCTGA**G**GC**C**CTGCA**G**GCC**C**TGAG | *ANKRD24* |
| 4 | OTC175-C15 | G**T**TGAAGC**G**CTGCA**TC**CCGTTGG | *ACP2* |
| 4 | OTC175-C16 | GCTG**G**AGCACTGCA**G**GC**T**G**C**TGG | *RABGGTA* |
| 4 | OTC175-C17 | GC**G**GAAGC**T**CTGC**C**CGC**A**GTCGG | *ZNF783* |
| 4 | OTC175-C18 | GCTGAAG**G**ACT**T**C**C**CGC**A**GTCGG | *ZNF358* |
| 4 | OTC175-C19 | GCTG**C**AGCACTGC**G**C**A**CCG**C**AGG | *KLHL34* |
| 4 | OTC175-C20 | GCTGAAGC**T**CTGC**G**CG**G**C**T**TTAG | *TTC25* |

OTC indicates an off-target candidate. Mismatches from the target sequence (20-nt gRNA175 hybrid region and 3-nt PAM sequence) are bolded and underlined.
