# Supplementary material for: High-Efficiency Targeted Editing of Large Viral Genomes by RNA-Guided Nucleases
Source: PLoS Pathog. 2014 May 1;10(5):e1004090. doi: 10.1371/journal.ppat.1004090 (PMC4006927; doi:10.1371/journal.ppat.1004090)
Supplement: Text S1 — Extended Materials and Methods. (DOC) [file ppat.1004090.s012.doc]

#### Text S1. Extended Materials and Methods

**Plasmid Construction**

The gRNAs were prepared by annealing oligo pairs (Table S1) and ligating into the BbsI site of gRNA cloning vectors (bicistronic expression vectors, plasmid 42230 and plasmid 42335; Addgene, Cambridge, Massachusetts, USA). pcw173, pcw174, pcw175, and pcw206 were generated by ligating the annealed dsDNA corresponding to gRNA-173, gRNA-174, gRNA-175, gRNA-206 into linearized plasmid 42230, respectively, whereas pcw178 and pcw180 were generated by ligating gRNA-173 and gRNA-175, respectively, into linearized plasmid 42335.

Donor plasmids pcw167 and pcw209 were constructed to express red fluorescent protein and EGFP protein, respectively. pcw167 was constructed by ligating the KpnI/NotI fragment from the pDsRed2-N1 vector (Clontech, Palo Alto, CA, USA), which encodes a red fluorescent protein derived from *Discosoma*, into the pShuttle-CMV vector (Agilent Technologies, Palo Alto, CA, USA). pcw209 was constructed by first amplifying the *TK* gene from the HSV1 genome using primers w042 and w043 (Table S1) and then ligating the fragment into the HindIII/EcoRI site of pCDNA3.1 (Life Technologies) to generate pcw021. The *EGFP* fragment was amplified from pEGFP-N2 (Clontech) using primers w286 and w287 and ligated into the BsiWI site of pcw201 to generate pcw209.

Plasmid pcw270 was constructed to provide additional XbaI restriction sites. To construct pcw270, two fragments from the pShuttle-CMV-EGFP vector were first PCR amplified using primers w340 and w341 for Fragment 1 and w342 and w343 for Fragment 2. Fragment 1 was digested with KpnI/XbaI and Fragment 2 was digested with XbaI/XhoI. These two fragments were then ligated into the vector pShuttle-CMV to generate pcw270.

The TALEN plasmids pcw277 and pcw278 were constructed by Obio Technology (Shanghai, China). The details of plasmid construction and the sequences for the TALEN plasmids are provided in the supplementary data.

**Transfection**

293FT cells were seeded into 6-well plates (Corning) at a density of 6×10^5^ cells/well 24 hours prior to transfection. The cells were transfected using Lipofectamine 2000 (Life Technologies) following the manufacturer’s recommended protocol. With no special instructions, for single plasmid transfection, 2 μg of plasmid was used, and for the co-expression of two plasmids, 3 μg of total plasmid was used.

**Western blot analysis**

The protein concentration of the cell lysate was determined using a protein assay reagent (Bio-Rad). An equal amount of total protein for each sample was analyzed by 8% SDS-PAGE. The separated proteins were transferred onto a polyvinylidene difluoride (PVDF) membrane using a semi-dry blotter (Bio-Rad). The membrane was blocked for 1 hour with 5% nonfat milk in TBS buffer (50 mmol/l Tris base, 150 mmol/l NaCl, pH 7.5) containing 0.05% Tween 20. After blocking, an anti-Flag antibody (M2 at a dilution of 1:500, Sigma-Aldrich, Shanghai, China) was added and incubated for 1 hour. The membrane was washed before an HRP-conjugated goat anti-mouse antibody (1:1000, Sigma-Aldrich) was added, and after incubation for 1 hour, the membrane was washed three times with TBS-Tween buffer. The specific signals were visualized using the ECL Plus Western blotting detection system (Pierce, Rockford, IL, USA).

**Indirect** **immunofluorescence assay (IFA)**

At 24 or 48 hours post-transfection, the cells were washed once with PBS and fixed in a 4% paraformaldehyde solution in PBS for 10 minutes. The cells were then treated with 0.1% Triton X-100 in PBS for 5 minutes and washed once with PBS. The samples were blocked with 1% BSA containing 1% donkey serum for 1 hour. Fixed cells were incubated for 1 hour with primary antibody (M2 at a dilution of 1:500, Sigma-Aldrich) followed by washing with PBS. A secondary antibody, Alexa Fluor 594 donkey anti-mouse (IgG+L) antibody (1:500, Sigma-Aldrich), was added, and the samples were incubated for 1 hour. The samples were then washed with PBS, and mounting medium was added for visualization on a fluorescent microscope (Nikon TE 2000-U; Nikon).

**Infection**

293FT cells were infected with ADV or HSV1 at an m.o.i. of 1-10 at 24 hours post-transfection. Total virus was harvested 36-48 hours after infection for SURVEYOR assay, viral titer, and restriction fragment length polymorphism (RFLP) analysis. AD293 cells were cultured to 80 to 90% confluence and then infected with P1 or P2’ to obtain P2 and P3’ for the SURVEYOR assay (Figure S3).

To determine the optimal post-transfection time, m.o.i., and harvest time, transfected 293FT cells were infected with ADV-EGFP at different time points (6-48 hours) and at different m.o.i. (0.1-100) and were harvested at different time points (12-84 hours).

**Plaque assay**

AD293 or Vero cells were seeded into 6-well plates at a density of 1×10^6^ cells/well 24 hours prior to infection. ADV or HSV1 were 10-fold serially diluted (1:1 to 1:10^8^) and then used to infect the AD293 or Vero cells, respectively. After a 6-hour incubation at 37°C, the viral supernatants were discarded, and fresh DMEM containing 0.4% UltraPure agarose (Life Technologies) and 2% FBS was added followed by incubation at 37ºC in 5% CO_2_. The plaques were counted at 4-7 days post-infection, and the results were recorded as the number of plaques per ml of inoculums. Fluorescent plaques were counted under a fluorescence microscope (Nikon TE 2000-U; Nikon, Tokyo, Japan). The G-2A filter was used for red fluorescence, B-2A for green fluorescence, and UV-2A for blue fluorescence (Figure 5C and 7G). Several replicates for each time point were used. Viral plaques were picked and grown in AD293 cells for ADV and in Vero cells for HSV1. After the plaques were picked, the agarose was removed, and the plaques were stained with DAPI (Vector Laboratories, Burlingame, USA). The collected viruses were harvested, and genomic DNA was extracted and amplified by PCR using primers prior to sending the results for sequencing as described above.

**Endpoint dilution assay**

Vero cells were seeded into 96-well plates 1 day prior to transfection at a density of 1x10^4^ cells/well. The Vero cells were infected with HSV1 at a 10-fold serial dilution (1:1 to 1:10^8^) with or without 100 μg/ml ACV. When CPE occurred, HSV1 from the highest dilution plate (single colonies) was collected for sequencing, and the number of positive and negative wells was recorded to calculate the TCID_50_ titer according to the Reed and Muench formula . The mutation rate of the *TK* gene was determined by comparing the difference between TCID_50_s in the presence or absence of ACV.

**Whole viral genome sequencing**

Adenovirus was amplified and purified using the Adeno-X™ Maxi Purification Kit (Clontech Laboratories, Takara Bio Inc., Beijing, China). The viral genome was extracted using the QIAamp MinElute Virus Spin Kit (QIAGEN, Shanghai, China) and submitted for sequencing (BGI) after PCR amplification. The primers used for PCR will be provided upon request.

**Reference**

1. Reed LJ, Muench H (1938) A simple method of estimating fifty percent endpoints. . Am J Hygiene 27: 493-497.
