# Supplementary material for: High-Efficiency Targeted Editing of Large Viral Genomes by RNA-Guided Nucleases
Source: PLoS Pathog. 2014 May 1;10(5):e1004090. doi: 10.1371/journal.ppat.1004090 (PMC4006927; doi:10.1371/journal.ppat.1004090)
Supplement: Text S2 — Supplementary sequences. (DOC) [file ppat.1004090.s013.doc]

**Text S2. Supplementary Sequences**

- U6-gRNA173

GAGGGCCTATTTCCCATGATTCCTTCATATTTGCATATACGATACAAGGCTGTTAGAGAGATAATTGGAATTAATTTGACTGTAAACACAAAGATATTAGTACAAAATACGTGACGTAGAAAGTAATAATTTCTTGGGTAGTTTGCAGTTTTAAAATTATGTTTTAAAATGGACTATCATATGCTTACCGTAACTTGAAAGTATTTCGATTTCTTGGCTTTATATATCTTGTGGAAAGGACGAAACACCGTGAACCGCATCGAGCTGAAGTTTTAGAGCTAGAAATAGCAAGTTAAAATAAGGCTAGTCCGTTATCAACTTGAAAAAGTGGCACCGAGTCGGTGCTTTTTT

- U6-gRNA174

GAGGGCCTATTTCCCATGATTCCTTCATATTTGCATATACGATACAAGGCTGTTAGAGAGATAATTGGAATTAATTTGACTGTAAACACAAAGATATTAGTACAAAATACGTGACGTAGAAAGTAATAATTTCTTGGGTAGTTTGCAGTTTTAAAATTATGTTTTAAAATGGACTATCATATGCTTACCGTAACTTGAAAGTATTTCGATTTCTTGGCTTTATATATCTTGTGGAAAGGACGAAACACCGGAGCGCACCATCTTCTTCAGTTTTAGAGCTAGAAATAGCAAGTTAAAATAAGGCTAGTCCGTTATCAACTTGAAAAAGTGGCACCGAGTCGGTGCTTTTTT

- U6-gRNA175

GAGGGCCTATTTCCCATGATTCCTTCATATTTGCATATACGATACAAGGCTGTTAGAGAGATAATTGGAATTAATTTGACTGTAAACACAAAGATATTAGTACAAAATACGTGACGTAGAAAGTAATAATTTCTTGGGTAGTTTGCAGTTTTAAAATTATGTTTTAAAATGGACTATCATATGCTTACCGTAACTTGAAAGTATTTCGATTTCTTGGCTTTATATATCTTGTGGAAAGGACGAAACACCGCTGAAGCACTGCACGCCGTGTTTTAGAGCTAGAAATAGCAAGTTAAAATAAGGCTAGTCCGTTATCAACTTGAAAAAGTGGCACCGAGTCGGTGCTTTTTT

- U6-gRNA206

GAGGGCCTATTTCCCATGATTCCTTCATATTTGCATATACGATACAAGGCTGTTAGAGAGATAATTGGAATTAATTTGACTGTAAACACAAAGATATTAGTACAAAATACGTGACGTAGAAAGTAATAATTTCTTGGGTAGTTTGCAGTTTTAAAATTATGTTTTAAAATGGACTATCATATGCTTACCGTAACTTGAAAGTATTTCGATTTCTTGGCTTTATATATCTTGTGGAAAGGACGAAACACCGAGGGCGCAACGCCGTACGTGTTTTAGAGCTAGAAATAGCAAGTTAAAATAAGGCTAGTCCGTTATCAACTTGAAAAAGTGGCACCGAGTCGGTGCTTTTTT

- EGFP_TALEN_Left CDS

ATGGACTACAAAGACCATGACGGTGATTATAAAGATCATGACATCGATTACAAGGATGACGATGACAAGATGGCCCCCAAGAAGAAGAGGAAGGTGGGCATTCACCGCGGGGTACCTATGGTGGACTTGAGGACACTCGGTTATTCGCAACAGCAACAGGAGAAAATCAAGCCTAAGGTCAGGAGCACCGTCGCGCAACACCACGAGGCGCTTGTGGGGCATGGCTTCACTCATGCGCATATTGTCGCGCTTTCACAGCACCCTGCGGCGCTTGGGACGGTGGCTGTCAAATACCAAGATATGATTGCGGCCCTGCCCGAAGCCACGCACGAGGCAATTGTAGGGGTCGGTAAACAGTGGTCGGGAGCGCGAGCACTTGAGGCGCTGCTGACTGTGGCGGGTGAGCTTAGGGGGCCTCCGCTCCAGCTCGACACCGGGCAGCTGCTGAAGATCGCGAAGAGAGGGGGAGTAACAGCGGTAGAGGCAGTGCACGCCTGGCGCAATGCGCTCACCGGGGCCCCCTTGAACCTGACCCCAGACCAGGTAGTCGCAATCGCGTCACATGACGGGGGAAAGCAAGCCCTGGAAACCGTGCAAAGGTTGTTGCCGGTCCTTTGTCAAGACCACGGCCTTACACCGGAGCAAGTCGTGGCCATTGCAAATAATAACGGTGGCAAACAGGCTCTTGAGACGGTTCAGAGACTTCTCCCAGTTCTCTGTCAAGCCCACGGGCTGACTCCCGATCAAGTTGTAGCGATTGCGTCCAACGGTGGAGGGAAACAAGCATTGGAGACTGTCCAACGGCTCCTTCCCGTGTTGTGTCAAGCCCACGGTTTGACGCCTGCACAAGTGGTCGCCATCGCCAACAACAACGGCGGTAAGCAGGCGCTGGAAACAGTACAGCGCCTGCTGCCTGTACTGTGCCAGGATCATGGACTGACCCCAGACCAGGTAGTCGCAATCGCGTCGAACATTGGGGGAAAGCAAGCCCTGGAAACCGTGCAAAGGTTGTTGCCGGTCCTTTGTCAAGACCACGGCCTTACACCGGAGCAAGTCGTGGCCATTGCATCCCACGACGGTGGCAAACAGGCTCTTGAGACGGTTCAGAGACTTCTCCCAGTTCTCTGTCAAGCCCACGGGCTGACTCCCGATCAAGTTGTAGCGATTGCGTCGCATGACGGAGGGAAACAAGCATTGGAGACTGTCCAACGGCTCCTTCCCGTGTTGTGTCAAGCCCACGGTTTGACGCCTGCACAAGTGGTCGCCATCGCCTCCAATATTGGCGGTAAGCAGGCGCTGGAAACAGTACAGCGCCTGCTGCCTGTACTGTGCCAGGATCATGGACTGACCCCAGACCAGGTAGTCGCAATCGCGTCACATGACGGGGGAAAGCAAGCCCTGGAAACCGTGCAAAGGTTGTTGCCGGTCCTTTGTCAAGACCACGGCCTTACACCGGAGCAAGTCGTGGCCATTGCATCCCACGACGGTGGCAAACAGGCTCTTGAGACGGTTCAGAGACTTCTCCCAGTTCTCTGTCAAGCCCACGGGCTGACTCCCGATCAAGTTGTAGCGATTGCGTCGCATGACGGAGGGAAACAAGCATTGGAGACTGTCCAACGGCTCCTTCCCGTGTTGTGTCAAGCCCACGGTTTGACGCCTGCACAAGTGGTCGCCATCGCCTCGAATGGCGGCGGTAAGCAGGCGCTGGAAACAGTACAGCGCCTGCTGCCTGTACTGTGCCAGGATCATGGACTGACCCCAGACCAGGTAGTCGCAATCGCGAACAATAATGGGGGAAAGCAAGCCCTGGAAACCGTGCAAAGGTTGTTGCCGGTCCTTTGTCAAGACCACGGCCTTACACCGGAGCAAGTCGTGGCCATTGCAAGCAACATCGGTGGCAAACAGGCTCTTGAGACGGTTCAGAGACTTCTCCCAGTTCTCTGTCAAGCCCACGGGCTGACTCCCGATCAAGTTGTAGCGATTGCGTCGCATGACGGAGGGAAACAAGCATTGGAGACTGTCCAACGGCTCCTTCCCGTGTTGTGTCAAGCCCACGGTTTGACGCCTGCACAAGTGGTCGCCATCGCCAGCCATGATGGCGGTAAGCAGGCGCTGGAAACAGTACAGCGCCTGCTGCCTGTACTGTGCCAGGATCATGGACTGACACCCGAACAGGTGGTCGCCATTGCTTCTAATGGGGGAGGACGGCCAGCCTTGGAGTCCATCGTAGCCCAATTGTCCAGGCCCGATCCCGCGTTGGCTGCGTTAACGAATGACCATCTGGTGGCGTTGGCATGTCTTGGTGGACGACCCGCGCTCGATGCAGTCAAAAAGGGTCTGCCTCATGCTCCCGCATTGATCAAAAGAACCAACCGGCGGATTCCCGAGAGAACTTCCCATCGAGTCGCGGGATCCCAACTAGTCAAAAGTGAACTGGAGGAGAAGAAATCTGAACTTCGTCATAAATTGAAATATGTGCCTCATGAATATATTGAATTAATTGAAATTGCCAGAAATTCCACTCAGGATAGAATTCTTGAAATGAAGGTAATGGAATTTTTTATGAAAGTTTATGGATATAGAGGTAAACATTTGGGTGGATCAAGGAAACCGGACGGAGCAATTTATACTGTCGGATCTCCTATTGATTACGGTGTGATCGTGGATACTAAAGCTTATAGCGGAGGTTATAATCTGCCAATTGGCCAAGCAGATGAAATGCAACGATATGTCGAAGAAAATCAAACACGAAACAAACATATCAACCCTAATGAATGGTGGAAAGTCTATCCATCTTCTGTAACGGAATTTAAGTTTTTATTTGTGAGTGGTCACTTTAAAGGAAACTACAAAGCTCAGCTTACACGATTAAATCATATCACTAATTGTAATGGAGCTGTTCTTAGTGTAGAAGAGCTTTTAATTGGTGGAGAAATGATTAAAGCCGGCACATTAACCTTAGAGGAAGTCAGACGGAAATTTAATAACGGCGAGATAAACTTTTAA

- EGFP_TALEN_Right CDS

ATGGACTACAAAGACCATGACGGTGATTATAAAGATCATGACATCGATTACAAGGATGACGATGACAAGATGGCCCCCAAGAAGAAGAGGAAGGTGGGCATTCACCGCGGGGTACCTATGGTGGACTTGAGGACACTCGGTTATTCGCAACAGCAACAGGAGAAAATCAAGCCTAAGGTCAGGAGCACCGTCGCGCAACACCACGAGGCGCTTGTGGGGCATGGCTTCACTCATGCGCATATTGTCGCGCTTTCACAGCACCCTGCGGCGCTTGGGACGGTGGCTGTCAAATACCAAGATATGATTGCGGCCCTGCCCGAAGCCACGCACGAGGCAATTGTAGGGGTCGGTAAACAGTGGTCGGGAGCGCGAGCACTTGAGGCGCTGCTGACTGTGGCGGGTGAGCTTAGGGGGCCTCCGCTCCAGCTCGACACCGGGCAGCTGCTGAAGATCGCGAAGAGAGGGGGAGTAACAGCGGTAGAGGCAGTGCACGCCTGGCGCAATGCGCTCACCGGGGCCCCCTTGAACCTGACCCCAGACCAGGTAGTCGCAATCGCGAACAATAATGGGGGAAAGCAAGCCCTGGAAACCGTGCAAAGGTTGTTGCCGGTCCTTTGTCAAGACCACGGCCTTACACCGGAGCAAGTCGTGGCCATTGCAAATAATAACGGTGGCAAACAGGCTCTTGAGACGGTTCAGAGACTTCTCCCAGTTCTCTGTCAAGCCCACGGGCTGACTCCCGATCAAGTTGTAGCGATTGCGTCCAACGGTGGAGGGAAACAAGCATTGGAGACTGTCCAACGGCTCCTTCCCGTGTTGTGTCAAGCCCACGGTTTGACGCCTGCACAAGTGGTCGCCATCGCCAGCCATGATGGCGGTAAGCAGGCGCTGGAAACAGTACAGCGCCTGCTGCCTGTACTGTGCCAGGATCATGGACTGACCCCAGACCAGGTAGTCGCAATCGCGAACAATAATGGGGGAAAGCAAGCCCTGGAAACCGTGCAAAGGTTGTTGCCGGTCCTTTGTCAAGACCACGGCCTTACACCGGAGCAAGTCGTGGCCATTGCAAATAATAACGGTGGCAAACAGGCTCTTGAGACGGTTCAGAGACTTCTCCCAGTTCTCTGTCAAGCCCACGGGCTGACTCCCGATCAAGTTGTAGCGATTGCGAATAACAATGGAGGGAAACAAGCATTGGAGACTGTCCAACGGCTCCTTCCCGTGTTGTGTCAAGCCCACGGTTTGACGCCTGCACAAGTGGTCGCCATCGCCAACAACAACGGCGGTAAGCAGGCGCTGGAAACAGTACAGCGCCTGCTGCCTGTACTGTGCCAGGATCATGGACTGACCCCAGACCAGGTAGTCGCAATCGCGTCAAACGGAGGGGGAAAGCAAGCCCTGGAAACCGTGCAAAGGTTGTTGCCGGTCCTTTGTCAAGACCACGGCCTTACACCGGAGCAAGTCGTGGCCATTGCAAGCAACATCGGTGGCAAACAGGCTCTTGAGACGGTTCAGAGACTTCTCCCAGTTCTCTGTCAAGCCCACGGGCTGACTCCCGATCAAGTTGTAGCGATTGCGAATAACAATGGAGGGAAACAAGCATTGGAGACTGTCCAACGGCTCCTTCCCGTGTTGTGTCAAGCCCACGGTTTGACGCCTGCACAAGTGGTCGCCATCGCCAGCCATGATGGCGGTAAGCAGGCGCTGGAAACAGTACAGCGCCTGCTGCCTGTACTGTGCCAGGATCATGGACTGACCCCAGACCAGGTAGTCGCAATCGCGAACAATAATGGGGGAAAGCAAGCCCTGGAAACCGTGCAAAGGTTGTTGCCGGTCCTTTGTCAAGACCACGGCCTTACACCGGAGCAAGTCGTGGCCATTGCAAATAATAACGGTGGCAAACAGGCTCTTGAGACGGTTCAGAGACTTCTCCCAGTTCTCTGTCAAGCCCACGGGCTGACTCCCGATCAAGTTGTAGCGATTGCGTCGCATGACGGAGGGAAACAAGCATTGGAGACTGTCCAACGGCTCCTTCCCGTGTTGTGTCAAGCCCACGGTCTGACACCCGAACAGGTGGTCGCCATTGCTTCTAATGGGGGAGGACGGCCAGCCTTGGAGTCCATCGTAGCCCAATTGTCCAGGCCCGATCCCGCGTTGGCTGCGTTAACGAATGACCATCTGGTGGCGTTGGCATGTCTTGGTGGACGACCCGCGCTCGATGCAGTCAAAAAGGGTCTGCCTCATGCTCCCGCATTGATCAAAAGAACCAACCGGCGGATTCCCGAGAGAACTTCCCATCGAGTCGCGGGATCCCAACTAGTCAAAAGTGAACTGGAGGAGAAGAAATCTGAACTTCGTCATAAATTGAAATATGTGCCTCATGAATATATTGAATTAATTGAAATTGCCAGAAATTCCACTCAGGATAGAATTCTTGAAATGAAGGTAATGGAATTTTTTATGAAAGTTTATGGATATAGAGGTAAACATTTGGGTGGATCAAGGAAACCGGACGGAGCAATTTATACTGTCGGATCTCCTATTGATTACGGTGTGATCGTGGATACTAAAGCTTATAGCGGAGGTTATAATCTGCCAATTGGCCAAGCAGATGAAATGCAACGATATGTCGAAGAAAATCAAACACGAAACAAACATATCAACCCTAATGAATGGTGGAAAGTCTATCCATCTTCTGTAACGGAATTTAAGTTTTTATTTGTGAGTGGTCACTTTAAAGGAAACTACAAAGCTCAGCTTACACGATTAAATCATATCACTAATTGTAATGGAGCTGTTCTTAGTGTAGAAGAGCTTTTAATTGGTGGAGAAATGATTAAAGCCGGCACATTAACCTTAGAGGAAGTCAGACGGAAATTTAATAACGGCGAGATAAACTTTTAA

- ADV-EGFP complete genome sequence


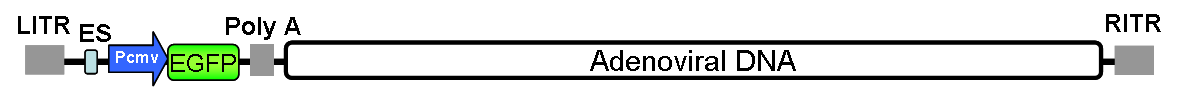


CATCATCAATAATATACCTTATTTTGGATTGAAGCCAATATGATAATGAGGGGGTGGAGTTTGTGACGTGGCGCGGGGCGTGGGAACGGGGCGGGTGACGTAGTAGTGTGGCGGAAGTGTGATGTTGCAAGTGTGGCGGAACACATGTAAGCGACGGATGTGGCAAAAGTGACGTTTTTGGTGTGCGCCGGTGTACACAGGAAGTGACAATTTTCGCGCGGTTTTAGGCGGATGTTGTAGTAAATTTGGGCGTAACCGAGTAAGATTTGGCCATTTTCGCGGGAAAACTGAATAAGAGGAAGTGAAATCTGAATAATTTTGTGTTACTCATAGCGCGTAATACTGTAATAGTAATCAATTACGGGGTCATTAGTTCATAGCCCATATATGGAGTTCCGCGTTACATAACTTACGGTAAATGGCCCGCCTGGCTGACCGCCCAACGACCCCCGCCCATTGACGTCAATAATGACGTATGTTCCCATAGTAACGCCAATAGGGACTTTCCATTGACGTCAATGGGTGGAGTATTTACGGTAAACTGCCCACTTGGCAGTACATCAAGTGTATCATATGCCAAGTACGCCCCCTATTGACGTCAATGACGGTAAATGGCCCGCCTGGCATTATGCCCAGTACATGACCTTATGGGACTTTCCTACTTGGCAGTACATCTACGTATTAGTCATCGCTATTACCATGGTGATGCGGTTTTGGCAGTACATCAATGGGCGTGGATAGCGGTTTGACTCACGGGGATTTCCAAGTCTCCACCCCATTGACGTCAATGGGAGTTTGTTTTGGCACCAAAATCAACGGGACTTTCCAAAATGTCGTAACAACTCCGCCCCATTGACGCAAATGGGCGGTAGGCGTGTACGGTGGGAGGTCTATATAAGCAGAGCTGGTTTAGTGAACCGTCAGATCCGCTAGAGATCTGGTACCGCGGGCCCGGGATCCACCGGCCGGTCGCCACCATGGTGAGCAAGGGCGAGGAGCTGTTCACCGGGGTGGTGCCCATCCTGGTCGAGCTGGACGGCGACGTAAACGGCCACAAGTTCAGCGTGTCCGGCGAGGGCGAGGGCGATGCCACCTACGGCAAGCTGACCCTGAAGTTCATCTGCACCACCGGCAAGCTGCCCGTGCCCTGGCCCACCCTCGTGACCACCCTGACCTACGGCGTGCAGTGCTTCAGCCGCTACCCCGACCACATGAAGCAGCACGACTTCTTCAAGTCCGCCATGCCCGAAGGCTACGTCCAGGAGCGCACCATCTTCTTCAAGGACGACGGCAACTACAAGACCCGCGCCGAGGTGAAGTTCGAGGGCGACACCCTGGTGAACCGCATCGAGCTGAAGGGCATCGACTTCAAGGAGGACGGCAACATCCTGGGGCACAAGCTGGAGTACAACTACAACAGCCACAACGTCTATATCATGGCCGACAAGCAGAAGAACGGCATCAAGGTGAACTTCAAGATCCGCCACAACATCGAGGACGGCAGCGTGCAGCTCGCCGACCACTACCAGCAGAACACCCCCATCGGCGACGGCCCCGTGCTGCTGCCCGACAACCACTACCTGAGCACCCAGTCCGCCCTGAGCAAAGACCCCAACGAGAAGCGCGATCACATGGTCCTGCTGGAGTTCGTGACCGCCGCCGGGATCACTCTCGGCATGGACGAGCTGTACAAGTAAAGCGGCCGCTCGAGCCTAAGCTTCTAGATAAGATATCCGATCCACCGGATCTAGATAACTGATCATAATCAGCCATACCACATTTGTAGAGGTTTTACTTGCTTTAAAAAACCTCCCACACCTCCCCCTGAACCTGAAACATAAAATGAATGCAATTGTTGTTGTTAACTTGTTTATTGCAGCTTATAATGGTTACAAATAAAGCAATAGCATCACAAATTTCACAAATAAAGCATTTTTTTCACTGCATTCTAGTTGTGGTTTGTCCAAACTCATCAATGTATCTTAACGCGGATCTGGGCGTGGTTAAGGGTGGGAAAGAATATATAAGGTGGGGGTCTTATGTAGTTTTGTATCTGTTTTGCAGCAGCCGCCGCCGCCATGAGCACCAACTCGTTTGATGGAAGCATTGTGAGCTCATATTTGACAACGCGCATGCCCCCATGGGCCGGGGTGCGTCAGAATGTGATGGGCTCCAGCATTGATGGTCGCCCCGTCCTGCCCGCAAACTCTACTACCTTGACCTACGAGACCGTGTCTGGAACGCCGTTGGAGACTGCAGCCTCCGCCGCCGCTTCAGCCGCTGCAGCCACCGCCCGCGGGATTGTGACTGACTTTGCTTTCCTGAGCCCGCTTGCAAGCAGTGCAGCTTCCCGTTCATCCGCCCGCGATGACAAGTTGACGGCTCTTTTGGCACAATTGGATTCTTTGACCCGGGAACTTAATGTCGTTTCTCAGCAGCTGTTGGATCTGCGCCAGCAGGTTTCTGCCCTGAAGGCTTCCTCCCCTCCCAATGCGGTTTAAAACATAAATAAAAAACCAGACTCTGTTTGGATTTGGATCAAGCAAGTGTCTTGCTGTCTTTATTTAGGGGTTTTGCGCGCGCGGTAGGCCCGGGACCAGCGGTCTCGGTCGTTGAGGGTCCTGTGTATTTTTTCCAGGACGTGGTAAAGGTGACTCTGGATGTTCAGATACATGGGCATAAGCCCGTCTCTGGGGTGGAGGTAGCACCACTGCAGAGCTTCATGCTGCGGGGTGGTGTTGTAGATGATCCAGTCGTAGCAGGAGCGCTGGGCGTGGTGCCTAAAAATGTCTTTCAGTAGCAAGCTGATTGCCAGGGGCAGGCCCTTGGTGTAAGTGTTTACAAAGCGGTTAAGCTGGGATGGGTGCATACGTGGGGATATGAGATGCATCTTGGACTGTATTTTTAGGTTGGCTATGTTCCCAGCCATATCCCTCCGGGGATTCATGTTGTGCAGAACCACCAGCACAGTGTATCCGGTGCACTTGGGAAATTTGTCATGTAGCTTAGAAGGAAATGCGTGGAAGAACTTGGAGACGCCCTTGTGACCTCCAAGATTTTCCATGCATTCGTCCATAATGATGGCAATGGGCCCACGGGCGGCGGCCTGGGCGAAGATATTTCTGGGATCACTAACGTCATAGTTGTGTTCCAGGATGAGATCGTCATAGGCCATTTTTACAAAGCGCGGGCGGAGGGTGCCAGACTGCGGTATAATGGTTCCATCCGGCCCAGGGGCGTAGTTACCCTCACAGATTTGCATTTCCCACGCTTTGAGTTCAGATGGGGGGATCATGTCTACCTGCGGGGCGATGAAGAAAACGGTTTCCGGGGTAGGGGAGATCAGCTGGGAAGAAAGCAGGTTCCTGAGCAGCTGCGACTTACCGCAGCCGGTGGGCCCGTAAATCACACCTATTACCGGCTGCAACTGGTAGTTAAGAGAGCTGCAGCTGCCGTCATCCCTGAGCAGGGGGGCCACTTCGTTAAGCATGTCCCTGACTCGCATGTTTTCCCTGACCAAATCCGCCAGAAGGCGCTCGCCGCCCAGCGATAGCAGTTCTTGCAAGGAAGCAAAGTTTTTCAACGGTTTGAGACCGTCCGCCGTAGGCATGCTTTTGAGCGTTTGACCAAGCAGTTCCAGGCGGTCCCACAGCTCGGTCACCTGCTCTACGGCATCTCGATCCAGCATATCTCCTCGTTTCGCGGGTTGGGGCGGCTTTCGCTGTACGGCAGTAGTCGGTGCTCGTCCAGACGGGCCAGGGTCATGTCTTTCCACGGGCGCAGGGTCCTCGTCAGCGTAGTCTGGGTCACGGTGAAGGGGTGCGCTCCGGGCTGCGCGCTGGCCAGGGTGCGCTTGAGGCTGGTCCTGCTGGTGCTGAAGCGCTGCCGGTCTTCGCCCTGCGCGTCGGCCAGGTAGCATTTGACCATGGTGTCATAGTCCAGCCCCTCCGCGGCGTGGCCCTTGGCGCGCAGCTTGCCCTTGGAGGAGGCGCCGCACGAGGGGCAGTGCAGACTTTTGAGGGCGTAGAGCTTGGGCGCGAGAAATACCGATTCCGGGGAGTAGGCATCCGCGCCGCAGGCCCCGCAGACGGTCTCGCATTCCACGAGCCAGGTGAGCTCTGGCCGTTCGGGGTCAAAAACCAGGTTTCCCCCATGCTTTTTGATGCGTTTCTTACCTCTGGTTTCCATGAGCCGGTGTCCACGCTCGGTGACGAAAAGGCTGTCCGTGTCCCCGTATACAGACTTGAGAGGCCTGTCCTCGAGCGGTGTTCCGCGGTCCTCCTCGTATAGAAACTCGGACCACTCTGAGACAAAGGCTCGCGTCCAGGCCAGCACGAAGGAGGCTAAGTGGGAGGGGTAGCGGTCGTTGTCCACTAGGGGGTCCACTCGCTCCAGGGTGTGAAGACACATGTCGCCCTCTTCGGCATCAAGGAAGGTGATTGGTTTGTAGGTGTAGGCCACGTGACCGGGTGTTCCTGAAGGGGGGCTATAAAAGGGGGTGGGGGCGCGTTCGTCCTCACTCTCTTCCGCATCGCTGTCTGCGAGGGCCAGCTGTTGGGGTGAGTACTCCCTCTGAAAAGCGGGCATGACTTCTGCGCTAAGATTGTCAGTTTCCAAAAACGAGGAGGATTTGATATTCACCTGGCCCGCGGTGATGCCTTTGAGGGTGGCCGCATCCATCTGGTCAGAAAAGACAATCTTTTTGTTGTCAAGCTTGGTGGCAAACGACCCGTAGAGGGCGTTGGACAGCAACTTGGCGATGGAGCGCAGGGTTTGGTTTTTGTCGCGATCGGCGCGCTCCTTGGCCGCGATGTTTAGCTGCACGTATTCGCGCGCAACGCACCGCCATTCGGGAAAGACGGTGGTGCGCTCGTCGGGCACCAGGTGCACGCGCCAACCGCGGTTGTGCAGGGTGACAAGGTCAACGCTGGTGGCTACCTCTCCGCGTAGGCGCTCGTTGGTCCAGCAGAGGCGGCCGCCCTTGCGCGAGCAGAATGGCGGTAGGGGGTCTAGCTGCGTCTCGTCCGGGGGGTCTGCGTCCACGGTAAAGACCCCGGGCAGCAGGCGCGCGTCGAAGTAGTCTATCTTGCATCCTTGCAAGTCTAGCGCCTGCTGCCATGCGCGGGCGGCAAGCGCGCGCTCGTATGGGTTGAGTGGGGGACCCCATGGCATGGGGTGGGTGAGCGCGGAGGCGTACATGCCGCAAATGTCGTAAACGTAGAGGGGCTCTCTGAGTATTCCAAGATATGTAGGGTAGCATCTTCCACCGCGGATGCTGGCGCGCACGTAATCGTATAGTTCGTGCGAGGGAGCGAGGAGGTCGGGACCGAGGTTGCTACGGGCGGGCTGCTCTGCTCGGAAGACTATCTGCCTGAAGATGGCATGTGAGTTGGATGATATGGTTGGACGCTGGAAGACGTTGAAGCTGGCGTCTGTGAGACCTACCGCGTCACGCACGAAGGAGGCGTAGGAGTCGCGCAGCTTGTTGACCAGCTCGGCGGTGACCTGCACGTCTAGGGCGCAGTAGTCCAGGGTTTCCTTGATGATGTCATACTTATCCTGTCCCTTTTTTTTCCACAGCTCGCGGTTGAGGACAAACTCTTCGCGGTCTTTCCAGTACTCTTGGATCGGAAACCCGTCGGCCTCCGAACGGTAAGAGCCTAGCATGTAGAACTGGTTGACGGCCTGGTAGGCGCAGCATCCCTTTTCTACGGGTAGCGCGTATGCCTGCGCGGCCTTCCGGAGCGAGGTGTGGGTGAGCGCAAAGGTGTCCCTGACCATGACTTTGAGGTACTGGTATTTGAAGTCAGTGTCGTCGCATCCGCCCTGCTCCCAGAGCAAAAAGTCCGTGCGCTTTTTGGAACGCGGATTTGGCAGGGCGAAGGTGACATCGTTGAAGAGTATCTTTCCCGCGCGAGGCATAAAGTTGCGTGTGATGCGGAAGGGTCCCGGCACCTCGGAACGGTTGTTAATTACCTGGGCGGCGAGCACGATCTCGTCAAAGCCGTTGATGTTGTGGCCCACAATGTAAAGTTCCAAGAAGCGCGGGATGCCCTTGATGGAAGGCAATTTTTTAAGTTCCTCGTAGGTGAGCTCTTCAGGGGAGCTGAGCCCGTGCTCTGAAAGGGCCCAGTCTGCAAGATGAGGGTTGGAAGCGACGAATGAGCTCCACAGGTCACGGGCCATTAGCATTTGCAGGTGGTCGCGAAAGGTCCTAAACTGGCGACCTATGGCCATTTTTTCTGGGGTGATGCAGTAGAAGGTAAGCGGGTCTTGTTCCCAGCGGTCCCATCCAAGGTTCGCGGCTAGGTCTCGCGCGGCAGTCACTAGAGGCTCATCTCCGCCGAACTTCATGACCAGCATGAAGGGCACGAGCTGCTTCCCAAAGGCCCCCATCCAAGTATAGGTCTCTACATCGTAGGTGACAAAGAGACGCTCGGTGCGAGGATGCGAGCCGATCGGGAAGAACTGGATCTCCCGCCACCAATTGGAGGAGTGGCTATTGATGTGGTGAAAGTAGAAGTCCCTGCGACGGGCCGAACACTCGTGCTGGCTTTTGTAAAAACGTGCGCAGTACTGGCAGCGGTGCACGGGCTGTACATCCTGCACGAGGTTGACCTGACGACCGCGCACAAGGAAGCAGAGTGGGAATTTGAGCCCCTCGCCTGGCGGGTTTGGCTGGTGGTCTTCTACTTCGGCTGCTTGTCCTTGACCGTCTGGCTGCTCGAGGGGAGTTACGGTGGATCGGACCACCACGCCGCGCGAGCCCAAAGTCCAGATGTCCGCGCGCGGCGGTCGGAGCTTGATGACAACATCGCGCAGATGGGAGCTGTCCATGGTCTGGAGCTCCCGCGGCGTCAGGTCAGGCGGGAGCTCCTGCAGGTTTACCTCGCATAGACGGGTCAGGGCGCGGGCTAGATCCAGGTGATACCTAATTTCCAGGGGCTGGTTGGTGGCGGCGTCGATGGCTTGCAAGAGGCCGCATCCCCGCGGCGCGACTACGGTACCGCGCGGCGGGCGGTGGGCCGCGGGGGTGTCCTTGGATGATGCATCTAAAAGCGGTGACGCGGGCGAGCCCCCGGAGGTAGGGGGGGCTCCGGACCCGCCGGGAGAGGGGGCAGGGGCACGTCGGCGCCGCGCGCGGGCAGGAGCTGGTGCTGCGCGCGTAGGTTGCTGGCGAACGCGACGACGCGGCGGTTGATCTCCTGAATCTGGCGCCTCTGCGTGAAGACGACGGGCCCGGTGAGCTTGAACCTGAAAGAGAGTTCGACAGAATCAATTTCGGTGTCGTTGACGGCGGCCTGGCGCAAAATCTCCTGCACGTCTCCTGAGTTGTCTTGATAGGCGATCTCGGCCATGAACTGCTCGATCTCTTCCTCCTGGAGATCTCCGCGTCCGGCTCGCTCCACGGTGGCGGCGAGGTCGTTGGAAATGCGGGCCATGAGCTGCGAGAAGGCGTTGAGGCCTCCCTCGTTCCAGACGCGGCTGTAGACCACGCCCCCTTCGGCATCGCGGGCGCGCATGACCACCTGCGCGAGATTGAGCTCCACGTGCCGGGCGAAGACGGCGTAGTTTCGCAGGCGCTGAAAGAGGTAGTTGAGGGTGGTGGCGGTGTGTTCTGCCACGAAGAAGTACATAACCCAGCGTCGCAACGTGGATTCGTTGATATCCCCCAAGGCCTCAAGGCGCTCCATGGCCTCGTAGAAGTCCACGGCGAAGTTGAAAAACTGGGAGTTGCGCGCCGACACGGTTAACTCCTCCTCCAGAAGACGGATGAGCTCGGCGACAGTGTCGCGCACCTCGCGCTCAAAGGCTACAGGGGCCTCTTCTTCTTCTTCAATCTCCTCTTCCATAAGGGCCTCCCCTTCTTCTTCTTCTGGCGGCGGTGGGGGAGGGGGGACACGGCGGCGACGACGGCGCACCGGGAGGCGGTCGACAAAGCGCTCGATCATCTCCCCGCGGCGACGGCGCATGGTCTCGGTGACGGCGCGGCCGTTCTCGCGGGGGCGCAGTTGGAAGACGCCGCCCGTCATGTCCCGGTTATGGGTTGGCGGGGGGCTGCCATGCGGCAGGGATACGGCGCTAACGATGCATCTCAACAATTGTTGTGTAGGTACTCCGCCGCCGAGGGACCTGAGCGAGTCCGCATCGACCGGATCGGAAAACCTCTCGAGAAAGGCGTCTAACCAGTCACAGTCGCAAGGTAGGCTGAGCACCGTGGCGGGCGGCAGCGGGCGGCGGTCGGGGTTGTTTCTGGCGGAGGTGCTGCTGATGATGTAATTAAAGTAGGCGGTCTTGAGACGGCGGATGGTCGACAGAAGCACCATGTCCTTGGGTCCGGCCTGCTGAATGCGCAGGCGGTCGGCCATGCCCCAGGCTTCGTTTTGACATCGGCGCAGGTCTTTGTAGTAGTCTTGCATGAGCCTTTCTACCGGCACTTCTTCTTCTCCTTCCTCTTGTCCTGCATCTCTTGCATCTATCGCTGCGGCGGCGGCGGAGTTTGGCCGTAGGTGGCGCCCTCTTCCTCCCATGCGTGTGACCCCGAAGCCCCTCATCGGCTGAAGCAGGGCTAGGTCGGCGACAACGCGCTCGGCTAATATGGCCTGCTGCACCTGCGTGAGGGTAGACTGGAAGTCATCCATGTCCACAAAGCGGTGGTATGCGCCCGTGTTGATGGTGTAAGTGCAGTTGGCCATAACGGACCAGTTAACGGTCTGGTGACCCGGCTGCGAGAGCTCGGTGTACCTGAGACGCGAGTAAGCCCTCGAGTCAAATACGTAGTCGTTGCAAGTCCGCACCAGGTACTGGTATCCCACCAAAAAGTGCGGCGGCGGCTGGCGGTAGAGGGGCCAGCGTAGGGTGGCCGGGGCTCCGGGGGCGAGATCTTCCAACATAAGGCGATGATATCCGTAGATGTACCTGGACATCCAGGTGATGCCGGCGGCGGTGGTGGAGGCGCGCGGAAAGTCGCGGACGCGGTTCCAGATGTTGCGCAGCGGCAAAAAGTGCTCCATGGTCGGGACGCTCTGGCCGGTCAGGCGCGCGCAATCGTTGACGCTCTAGCGTGCAAAAGGAGAGCCTGTAAGCGGGCACTCTTCCGTGGTCTGGTGGATAAATTCGCAAGGGTATCATGGCGGACGACCGGGGTTCGAGCCCCGTATCCGGCCGTCCGCCGTGATCCATGCGGTTACCGCCCGCGTGTCGAACCCAGGTGTGCGACGTCAGACAACGGGGGAGTGCTCCTTTTGGCTTCCTTCCAGGCGCGGCGGCTGCTGCGCTAGCTTTTTTGGCCACTGGCCGCGCGCAGCGTAAGCGGTTAGGCTGGAAAGCGAAAGCATTAAGTGGCTCGCTCCCTGTAGCCGGAGGGTTATTTTCCAAGGGTTGAGTCGCGGGACCCCCGGTTCGAGTCTCGGACCGGCCGGACTGCGGCGAACGGGGGTTTGCCTCCCCGTCATGCAAGACCCCGCTTGCAAATTCCTCCGGAAACAGGGACGAGCCCCTTTTTTGCTTTTCCCAGATGCATCCGGTGCTGCGGCAGATGCGCCCCCCTCCTCAGCAGCGGCAAGAGCAAGAGCAGCGGCAGACATGCAGGGCACCCTCCCCTCCTCCTACCGCGTCAGGAGGGGCGACATCCGCGGTTGACGCGGCAGCAGATGGTGATTACGAACCCCCGCGGCGCCGGGCCCGGCACTACCTGGACTTGGAGGAGGGCGAGGGCCTGGCGCGGCTAGGAGCGCCCTCTCCTGAGCGGCACCCAAGGGTGCAGCTGAAGCGTGATACGCGTGAGGCGTACGTGCCGCGGCAGAACCTGTTTCGCGACCGCGAGGGAGAGGAGCCCGAGGAGATGCGGGATCGAAAGTTCCACGCAGGGCGCGAGCTGCGGCATGGCCTGAATCGCGAGCGGTTGCTGCGCGAGGAGGACTTTGAGCCCGACGCGCGAACCGGGATTAGTCCCGCGCGCGCACACGTGGCGGCCGCCGACCTGGTAACCGCATACGAGCAGACGGTGAACCAGGAGATTAACTTTCAAAAAAGCTTTAACAACCACGTGCGTACGCTTGTGGCGCGCGAGGAGGTGGCTATAGGACTGATGCATCTGTGGGACTTTGTAAGCGCGCTGGAGCAAAACCCAAATAGCAAGCCGCTCATGGCGCAGCTGTTCCTTATAGTGCAGCACAGCAGGGACAACGAGGCATTCAGGGATGCGCTGCTAAACATAGTAGAGCCCGAGGGCCGCTGGCTGCTCGATTTGATAAACATCCTGCAGAGCATAGTGGTGCAGGAGCGCAGCTTGAGCCTGGCTGACAAGGTGGCCGCCATCAACTATTCCATGCTTAGCCTGGGCAAGTTTTACGCCCGCAAGATATACCATACCCCTTACGTTCCCATAGACAAGGAGGTAAAGATCGAGGGGTTCTACATGCGCATGGCGCTGAAGGTGCTTACCTTGAGCGACGACCTGGGCGTTTATCGCAACGAGCGCATCCACAAGGCCGTGAGCGTGAGCCGGCGGCGCGAGCTCAGCGACCGCGAGCTGATGCACAGCCTGCAAAGGGCCCTGGCTGGCACGGGCAGCGGCGATAGAGAGGCCGAGTCCTACTTTGACGCGGGCGCTGACCTGCGCTGGGCCCCAAGCCGACGCGCCCTGGAGGCAGCTGGGGCCGGACCTGGGCTGGCGGTGGCACCCGCGCGCGCTGGCAACGTCGGCGGCGTGGAGGAATATGACGAGGACGATGAGTACGAGCCAGAGGACGGCGAGTACTAAGCGGTGATGTTTCTGATCAGATGATGCAAGACGCAACGGACCCGGCGGTGCGGGCGGCGCTGCAGAGCCAGCCGTCCGGCCTTAACTCCACGGACGACTGGCGCCAGGTCATGGACCGCATCATGTCGCTGACTGCGCGCAATCCTGACGCGTTCCGGCAGCAGCCGCAGGCCAACCGGCTCTCCGCAATTCTGGAAGCGGTGGTCCCGGCGCGCGCAAACCCCACGCACGAGAAGGTGCTGGCGATCGTAAACGCGCTGGCCGAAAACAGGGCCATCCGGCCCGACGAGGCCGGCCTGGTCTACGACGCGCTGCTTCAGCGCGTGGCTCGTTACAACAGCGGCAACGTGCAGACCAACCTGGACCGGCTGGTGGGGGATGTGCGCGAGGCCGTGGCGCAGCGTGAGCGCGCGCAGCAGCAGGGCAACCTGGGCTCCATGGTTGCACTAAACGCCTTCCTGAGTACACAGCCCGCCAACGTGCCGCGGGGACAGGAGGACTACACCAACTTTGTGAGCGCACTGCGGCTAATGGTGACTGAGACACCGCAAAGTGAGGTGTACCAGTCTGGGCCAGACTATTTTTTCCAGACCAGTAGACAAGGCCTGCAGACCGTAAACCTGAGCCAGGCTTTCAAAAACTTGCAGGGGCTGTGGGGGGTGCGGGCTCCCACAGGCGACCGCGCGACCGTGTCTAGCTTGCTGACGCCCAACTCGCGCCTGTTGCTGCTGCTAATAGCGCCCTTCACGGACAGTGGCAGCGTGTCCCGGGACACATACCTAGGTCACTTGCTGACACTGTACCGCGAGGCCATAGGTCAGGCGCATGTGGACGAGCATACTTTCCAGGAGATTACAAGTGTCAGCCGCGCGCTGGGGCAGGAGGACACGGGCAGCCTGGAGGCAACCCTAAACTACCTGCTGACCAACCGGCGGCAGAAGATCCCCTCGTTGCACAGTTTAAACAGCGAGGAGGAGCGCATTTTGCGCTACGTGCAGCAGAGCGTGAGCCTTAACCTGATGCGCGACGGGGTAACGCCCAGCGTGGCGCTGGACATGACCGCGCGCAACATGGAACCGGGCATGTATGCCTCAAACCGGCCGTTTATCAACCGCCTAATGGACTACTTGCATCGCGCGGCCGCCGTGAACCCCGAGTATTTCACCAATGCCATCTTGAACCCGCACTGGCTACCGCCCCCTGGTTTCTACACCGGGGGATTCGAGGTGCCCGAGGGTAACGATGGATTCCTCTGGGACGACATAGACGACAGCGTGTTTTCCCCGCAACCGCAGACCCTGCTAGAGTTGCAACAGCGCGAGCAGGCAGAGGCGGCGCTGCGAAAGGAAAGCTTCCGCAGGCCAAGCAGCTTGTCCGATCTAGGCGCTGCGGCCCCGCGGTCAGATGCTAGTAGCCCATTTCCAAGCTTGATAGGGTCTCTTACCAGCACTCGCACCACCCGCCCGCGCCTGCTGGGCGAGGAGGAGTACCTAAACAACTCGCTGCTGCAGCCGCAGCGCGAAAAAAACCTGCCTCCGGCATTTCCCAACAACGGGATAGAGAGCCTAGTGGACAAGATGAGTAGATGGAAGACGTACGCGCAGGAGCACAGGGACGTGCCAGGCCCGCGCCCGCCCACCCGTCGTCAAAGGCACGACCGTCAGCGGGGTCTGGTGTGGGAGGACGATGACTCGGCAGACGACAGCAGCGTCCTGGATTTGGGAGGGAGTGGCAACCCGTTTGCGCACCTTCGCCCCAGGCTGGGGAGAATGTTTTAAAAAAAAAAAAGCATGATGCAAAATAAAAAACTCACCAAGGCCATGGCACCGAGCGTTGGTTTTCTTGTATTCCCCTTAGTATGCGGCGCGCGGCGATGTATGAGGAAGGTCCTCCTCCCTCCTACGAGAGTGTGGTGAGCGCGGCGCCAGTGGCGGCGGCGCTGGGTTCTCCCTTCGATGCTCCCCTGGACCCGCCGTTTGTGCCTCCGCGGTACCTGCGGCCTACCGGGGGGAGAAACAGCATCCGTTACTCTGAGTTGGCACCCCTATTCGACACCACCCGTGTGTACCTGGTGGACAACAAGTCAACGGATGTGGCATCCCTGAACTACCAGAACGACCACAGCAACTTTCTGACCACGGTCATTCAAAACAATGACTACAGCCCGGGGGAGGCAAGCACACAGACCATCAATCTTGACGACCGGTCGCACTGGGGCGGCGACCTGAAAACCATCCTGCATACCAACATGCCAAATGTGAACGAGTTCATGTTTACCAATAAGTTTAAGGCGCGGGTGATGGTGTCGCGCTTGCCTACTAAGGACAATCAGGTGGAGCTGAAATACGAGTGGGTGGAGTTCACGCTGCCCGAGGGCAACTACTCCGAGACCATGACCATAGACCTTATGAACAACGCGATCGTGGAGCACTACTTGAAAGTGGGCAGACAGAACGGGGTTCTGGAAAGCGACATCGGGGTAAAGTTTGACACCCGCAACTTCAGACTGGGGTTTGACCCCGTCACTGGTCTTGTCATGCCTGGGGTATATACAAACGAAGCCTTCCATCCAGACATCATTTTGCTGCCAGGATGCGGGGTGGACTTCACCCACAGCCGCCTGAGCAACTTGTTGGGCATCCGCAAGCGGCAACCCTTCCAGGAGGGCTTTAGGATCACCTACGATGATCTGGAGGGTGGTAACATTCCCGCACTGTTGGATGTGGACGCCTACCAGGCGAGCTTGAAAGATGACACCGAACAGGGCGGGGGTGGCGCAGGCGGCAGCAACAGCAGTGGCAGCGGCGCGGAAGAGAACTCCAACGCGGCAGCCGCGGCAATGCAGCCGGTGGAGGACATGAACGATCATGCCATTCGCGGCGACACCTTTGCCACACGGGCTGAGGAGAAGCGCGCTGAGGCCGAAGCAGCGGCCGAAGCTGCCGCCCCCGCTGCGCAACCCGAGGTCGAGAAGCCTCAGAAGAAACCGGTGATCAAACCCCTGACAGAGGACAGCAAGAAACGCAGTTACAACCTAATAAGCAATGACAGCACCTTCACCCAGTACCGCAGCTGGTACCTTGCATACAACTACGGCGACCCTCAGACCGGAATCCGCTCATGGACCCTGCTTTGCACTCCTGACGTAACCTGCGGCTCGGAGCAGGTCTACTGGTCGTTGCCAGACATGATGCAAGACCCCGTGACCTTCCGCTCCACGCGCCAGATCAGCAACTTTCCGGTGGTGGGCGCCGAGCTGTTGCCCGTGCACTCCAAGAGCTTCTACAACGACCAGGCCGTCTACTCCCAACTCATCCGCCAGTTTACCTCTCTGACCCACGTGTTCAATCGCTTTCCCGAGAACCAGATTTTGGCGCGCCCGCCAGCCCCCACCATCACCACCGTCAGTGAAAACGTTCCTGCTCTCACAGATCACGGGACGCTACCGCTGCGCAACAGCATCGGAGGAGTCCAGCGAGTGACCATTACTGACGCCAGACGCCGCACCTGCCCCTACGTTTACAAGGCCCTGGGCATAGTCTCGCCGCGCGTCCTATCGAGCCGCACTTTTTGAGCAAGCATGTCCATCCTTATATCGCCCAGCAATAACACAGGCTGGGGCCTGCGCTTCCCAAGCAAGATGTTTGGCGGGGCCAAGAAGCGCTCCGACCAACACCCAGTGCGCGTGCGCGGGCACTACCGCGCGCCCTGGGGCGCGCACAAACGCGGCCGCACTGGGCGCACCACCGTCGATGACGCCATCGACGCGGTGGTGGAGGAGGCGCGCAACTACACGCCCACGCCGCCACCAGTGTCCACAGTGGACGCGGCCATTCAGACCGTGGTGCGCGGAGCCCGGCGCTATGCTAAAATGAAGAGACGGCGGAGGCGCGTAGCACGTCGCCACCGCCGCCGACCCGGCACTGCCGCCCAACGCGCGGCGGCGGCCCTGCTTAACCGCGCACGTCGCACCGGCCGACGGGCGGCCATGCGGGCCGCTCGAAGGCTGGCCGCGGGTATTGTCACTGTGCCCCCCAGGTCCAGGCGACGAGCGGCCGCCGCAGCAGCCGCGGCCATTAGTGCTATGACTCAGGGTCGCAGGGGCAACGTGTATTGGGTGCGCGACTCGGTTAGCGGCCTGCGCGTGCCCGTGCGCACCCGCCCCCCGCGCAACTAGATTGCAAGAAAAAACTACTTAGACTCGTACTGTTGTATGTATCCAGCGGCGGCGGCGCGCAACGAAGCTATGTCCAAGCGCAAAATCAAAGAAGAGATGCTCCAGGTCATCGCGCCGGAGATCTATGGCCCCCCGAAGAAGGAAGAGCAGGATTACAAGCCCCGAAAGCTAAAGCGGGTCAAAAAGAAAAAGAAAGATGATGATGATGAACTTGACGACGAGGTGGAACTGCTGCACGCTACCGCGCCCAGGCGACGGGTACAGTGGAAAGGTCGACGCGTAAAACGTGTTTTGCGACCCGGCACCACCGTAGTCTTTACGCCCGGTGAGCGCTCCACCCGCACCTACAAGCGCGTGTATGATGAGGTGTACGGCGACGAGGACCTGCTTGAGCAGGCCAACGAGCGCCTCGGGGAGTTTGCCTACGGAAAGCGGCATAAGGACATGCTGGCGTTGCCGCTGGACGAGGGCAACCCAACACCTAGCCTAAAGCCCGTAACACTGCAGCAGGTGCTGCCCGCGCTTGCACCGTCCGAAGAAAAGCGCGGCCTAAAGCGCGAGTCTGGTGACTTGGCACCCACCGTGCAGCTGATGGTACCCAAGCGCCAGCGACTGGAAGATGTCTTGGAAAAAATGACCGTGGAACCTGGGCTGGAGCCCGAGGTCCGCGTGCGGCCAATCAAGCAGGTGGCGCCGGGACTGGGCGTGCAGACCGTGGACGTTCAGATACCCACTACCAGTAGCACCAGTATTGCCACCGCCACAGAGGGCATGGAGACACAAACGTCCCCGGTTGCCTCAGCGGTGGCGGATGCCGCGGTGCAGGCGGTCGCTGCGGCCGCGTCCAAGACCTCTACGGAGGTGCAAACGGACCCGTGGATGTTTCGCGTTTCAGCCCCCCGGCGCCCGCGCCGTTCGAGGAAGTACGGCGCCGCCAGCGCGCTACTGCCCGAATATGCCCTACATCCTTCCATTGCGCCTACCCCCGGCTATCGTGGCTACACCTACCGCCCCAGAAGACGAGCAACTACCCGACGCCGAACCACCACTGGAACCCGCCGCCGCCGTCGCCGTCGCCAGCCCGTGCTGGCCCCGATTTCCGTGCGCAGGGTGGCTCGCGAAGGAGGCAGGACCCTGGTGCTGCCAACAGCGCGCTACCACCCCAGCATCGTTTAAAAGCCGGTCTTTGTGGTTCTTGCAGATATGGCCCTCACCTGCCGCCTCCGTTTCCCGGTGCCGGGATTCCGAGGAAGAATGCACCGTAGGAGGGGCATGGCCGGCCACGGCCTGACGGGCGGCATGCGTCGTGCGCACCACCGGCGGCGGCGCGCGTCGCACCGTCGCATGCGCGGCGGTATCCTGCCCCTCCTTATTCCACTGATCGCCGCGGCGATTGGCGCCGTGCCCGGAATTGCATCCGTGGCCTTGCAGGCGCAGAGACACTGATTAAAAACAAGTTGCATGTGGAAAAATCAAAATAAAAAGTCTGGACTCTCACGCTCGCTTGGTCCTGTAACTATTTTGTAGAATGGAAGACATCAACTTTGCGTCTCTGGCCCCGCGACACGGCTCGCGCCCGTTCATGGGAAACTGGCAAGATATCGGCACCAGCAATATGAGCGGTGGCGCCTTCAGCTGGGGCTCGCTGTGGAGCGGCATTAAAAATTTCGGTTCCACCGTTAAGAACTATGGCAGCAAGGCCTGGAACAGCAGCACAGGCCAGATGCTGAGGGATAAGTTGAAAGAGCAAAATTTCCAACAAAAGGTGGTAGATGGCCTGGCCTCTGGCATTAGCGGGGTGGTGGACCTGGCCAACCAGGCAGTGCAAAATAAGATTAACAGTAAGCTTGATCCCCGCCCTCCCGTAGAGGAGCCTCCACCGGCCGTGGAGACAGTGTCTCCAGAGGGGCGTGGCGAAAAGCGTCCGCGCCCCGACAGGGAAGAAACTCTGGTGACGCAAATAGACGAGCCTCCCTCGTACGAGGAGGCACTAAAGCAAGGCCTGCCCACCACCCGTCCCATCGCGCCCATGGCTACCGGAGTGCTGGGCCAGCACACACCCGTAACGCTGGACCTGCCTCCCCCCGCCGACACCCAGCAGAAACCTGTGCTGCCAGGCCCGACCGCCGTTGTTGTAACCCGTCCTAGCCGCGCGTCCCTGCGCCGCGCCGCCAGCGGTCCGCGATCGTTGCGGCCCGTAGCCAGTGGCAACTGGCAAAGCACACTGAACAGCATCGTGGGTCTGGGGGTGCAATCCCTGAAGCGCCGACGATGCTTCTGATAGCTAACGTGTCGTATGTGTGTCATGTATGCGTCCATGTCGCCGCCAGAGGAGCTGCTGAGCCGCCGCGCGCCCGCTTTCCAAGATGGCTACCCCTTCGATGATGCCGCAGTGGTCTTACATGCACATCTCGGGCCAGGACGCCTCGGAGTACCTGAGCCCCGGGCTGGTGCAGTTTGCCCGCGCCACCGAGACGTACTTCAGCCTGAATAACAAGTTTAGAAACCCCACGGTGGCGCCTACGCACGACGTGACCACAGACCGGTCCCAGCGTTTGACGCTGCGGTTCATCCCTGTGGACCGTGAGGATACTGCGTACTCGTACAAGGCGCGGTTCACCCTAGCTGTGGGTGATAACCGTGTGCTGGACATGGCTTCCACGTACTTTGACATCCGCGGCGTGCTGGACAGGGGCCCTACTTTTAAGCCCTACTCTGGCACTGCCTACAACGCCCTGGCTCCCAAGGGTGCCCCAAATCCTTGCGAATGGGATGAAGCTGCTACTGCTCTTGAAATAAACCTAGAAGAAGAGGACGATGACAACGAAGACGAAGTAGACGAGCAAGCTGAGCAGCAAAAAACTCACGTATTTGGGCAGGCGCCTTATTCTGGTATAAATATTACAAAGGAGGGTATTCAAATAGGTGTCGAAGGTCAAACACCTAAATATGCCGATAAAACATTTCAACCTGAACCTCAAATAGGAGAATCTCAGTGGTACGAAACAGAAATTAATCATGCAGCTGGGAGAGTCCTAAAAAAGACTACCCCAATGAAACCATGTTACGGTTCATATGCAAAACCCACAAATGAAAATGGAGGGCAAGGCATTCTTGTAAAGCAACAAAATGGAAAGCTAGAAAGTCAAGTGGAAATGCAATTTTTCTCAACTACTGAGGCAGCCGCAGGCAATGGTGATAACTTGACTCCTAAAGTGGTATTGTACAGTGAAGATGTAGATATAGAAACCCCAGACACTCATATTTCTTACATGCCCACTATTAAGGAAGGTAACTCACGAGAACTAATGGGCCAACAATCTATGCCCAACAGGCCTAATTACATTGCTTTTAGGGACAATTTTATTGGTCTAATGTATTACAACAGCACGGGTAATATGGGTGTTCTGGCGGGCCAAGCATCGCAGTTGAATGCTGTTGTAGATTTGCAAGACAGAAACACAGAGCTTTCATACCAGCTTTTGCTTGATTCCATTGGTGATAGAACCAGGTACTTTTCTATGTGGAATCAGGCTGTTGACAGCTATGATCCAGATGTTAGAATTATTGAAAATCATGGAACTGAAGATGAACTTCCAAATTACTGCTTTCCACTGGGAGGTGTGATTAATACAGAGACTCTTACCAAGGTAAAACCTAAAACAGGTCAGGAAAATGGATGGGAAAAAGATGCTACAGAATTTTCAGATAAAAATGAAATAAGAGTTGGAAATAATTTTGCCATGGAAATCAATCTAAATGCCAACCTGTGGAGAAATTTCCTGTACTCCAACATAGCGCTGTATTTGCCCGACAAGCTAAAGTACAGTCCTTCCAACGTAAAAATTTCTGATAACCCAAACACCTACGACTACATGAACAAGCGAGTGGTGGCTCCCGGGCTAGTGGACTGCTACATTAACCTTGGAGCACGCTGGTCCCTTGACTATATGGACAACGTCAACCCATTTAACCACCACCGCAATGCTGGCCTGCGCTACCGCTCAATGTTGCTGGGCAATGGTCGCTATGTGCCCTTCCACATCCAGGTGCCTCAGAAGTTCTTTGCCATTAAAAACCTCCTTCTCCTGCCGGGCTCATACACCTACGAGTGGAACTTCAGGAAGGATGTTAACATGGTTCTGCAGAGCTCCCTAGGAAATGACCTAAGGGTTGACGGAGCCAGCATTAAGTTTGATAGCATTTGCCTTTACGCCACCTTCTTCCCCATGGCCCACAACACCGCCTCCACGCTTGAGGCCATGCTTAGAAACGACACCAACGACCAGTCCTTTAACGACTATCTCTCCGCCGCCAACATGCTCTACCCTATACCCGCCAACGCTACCAACGTGCCCATATCCATCCCCTCCCGCAACTGGGCGGCTTTCCGCGGCTGGGCCTTCACGCGCCTTAAGACTAAGGAAACCCCATCACTGGGCTCGGGCTACGACCCTTATTACACCTACTCTGGCTCTATACCCTACCTAGATGGAACCTTTTACCTCAACCACACCTTTAAGAAGGTGGCCATTACCTTTGACTCTTCTGTCAGCTGGCCTGGCAATGACCGCCTGCTTACCCCCAACGAGTTTGAAATTAAGCGCTCAGTTGACGGGGAGGGTTACAACGTTGCCCAGTGTAACATGACCAAAGACTGGTTCCTGGTACAAATGCTAGCTAACTATAACATTGGCTACCAGGGCTTCTATATCCCAGAGAGCTACAAGGACCGCATGTACTCCTTCTTTAGAAACTTCCAGCCCATGAGCCGTCAGGTGGTGGATGATACTAAATACAAGGACTACCAACAGGTGGGCATCCTACACCAACACAACAACTCTGGATTTGTTGGCTACCTTGCCCCCACCATGCGCGAAGGACAGGCCTACCCTGCTAACTTCCCCTATCCGCTTATAGGCAAGACCGCAGTTGACAGCATTACCCAGAAAAAGTTTCTTTGCGATCGCACCCTTTGGCGCATCCCATTCTCCAGTAACTTTATGTCCATGGGCGCACTCACAGACCTGGGCCAAAACCTTCTCTACGCCAACTCCGCCCACGCGCTAGACATGACTTTTGAGGTGGATCCCATGGACGAGCCCACCCTTCTTTATGTTTTGTTTGAAGTCTTTGACGTGGTCCGTGTGCACCAGCCGCACCGCGGCGTCATCGAAACCGTGTACCTGCGCACGCCCTTCTCGGCCGGCAACGCCACAACATAAAGAAGCAAGCAACATCAACAACAGCTGCCGCCATGGGCTCCAGTGAGCAGGAACTGAAAGCCATTGTCAAAGATCTTGGTTGTGGGCCATATTTTTTGGGCACCTATGACAAGCGCTTTCCAGGCTTTGTTTCTCCACACAAGCTCGCCTGCGCCATAGTCAATACGGCCGGTCGCGAGACTGGGGGCGTACACTGGATGGCCTTTGCCTGGAACCCGCACTCAAAAACATGCTACCTCTTTGAGCCCTTTGGCTTTTCTGACCAGCGACTCAAGCAGGTTTACCAGTTTGAGTACGAGTCACTCCTGCGCCGTAGCGCCATTGCTTCTTCCCCCGACCGCTGTATAACGCTGGAAAAGTCCACCCAAAGCGTACAGGGGCCCAACTCGGCCGCCTGTGGACTATTCTGCTGCATGTTTCTCCACGCCTTTGCCAACTGGCCCCAAACTCCCATGGATCACAACCCCACCATGAACCTTATTACCGGGGTACCCAACTCCATGCTCAACAGTCCCCAGGTACAGCCCACCCTGCGTCGCAACCAGGAACAGCTCTACAGCTTCCTGGAGCGCCACTCGCCCTACTTCCGCAGCCACAGTGCGCAGATTAGGAGCGCCACTTCTTTTTGTCACTTGAAAAACATGTAAAAATAATGTACTAGAGACACTTTCAATAAAGGCAAATGCTTTTATTTGTACACTCTCGGGTGATTATTTACCCCCACCCTTGCCGTCTGCGCCGTTTAAAAATCAAAGGGGTTCTGCCGCGCATCGCTATGCGCCACTGGCAGGGACACGTTGCGATACTGGTGTTTAGTGCTCCACTTAAACTCAGGCACAACCATCCGCGGCAGCTCGGTGAAGTTTTCACTCCACAGGCTGCGCACCATCACCAACGCGTTTAGCAGGTCGGGCGCCGATATCTTGAAGTCGCAGTTGGGGCCTCCGCCCTGCGCGCGCGAGTTGCGATACACAGGGTTGCAGCACTGGAACACTATCAGCGCCGGGTGGTGCACGCTGGCCAGCACGCTCTTGTCGGAGATCAGATCCGCGTCCAGGTCCTCCGCGTTGCTCAGGGCGAACGGAGTCAACTTTGGTAGCTGCCTTCCCAAAAAGGGCGCGTGCCCAGGCTTTGAGTTGCACTCGCACCGTAGTGGCATCAAAAGGTGACCGTGCCCGGTCTGGGCGTTAGGATACAGCGCCTGCATAAAAGCCTTGATCTGCTTAAAAGCCACCTGAGCCTTTGCGCCTTCAGAGAAGAACATGCCGCAAGACTTGCCGGAAAACTGATTGGCCGGACAGGCCGCGTCGTGCACGCAGCACCTTGCGTCGGTGTTGGAGATCTGCACCACATTTCGGCCCCACCGGTTCTTCACGATCTTGGCCTTGCTAGACTGCTCCTTCAGCGCGCGCTGCCCGTTTTCGCTCGTCACATCCATTTCAATCACGTGCTCCTTATTTATCATAATGCTTCCGTGTAGACACTTAAGCTCGCCTTCGATCTCAGCGCAGCGGTGCAGCCACAACGCGCAGCCCGTGGGCTCGTGATGCTTGTAGGTCACCTCTGCAAACGACTGCAGGTACGCCTGCAGGAATCGCCCCATCATCGTCACAAAGGTCTTGTTGCTGGTGAAGGTCAGCTGCAACCCGCGGTGCTCCTCGTTCAGCCAGGTCTTGCATACGGCCGCCAGAGCTTCCACTTGGTCAGGCAGTAGTTTGAAGTTCGCCTTTAGATCGTTATCCACGTGGTACTTGTCCATCAGCGCGCGCGCAGCCTCCATGCCCTTCTCCCACGCAGACACGATCGGCACACTCAGCGGGTTCATCACCGTAATTTCACTTTCCGCTTCGCTGGGCTCTTCCTCTTCCTCTTGCGTCCGCATACCACGCGCCACTGGGTCGTCTTCATTCAGCCGCCGCACTGTGCGCTTACCTCCTTTGCCATGCTTGATTAGCACCGGTGGGTTGCTGAAACCCACCATTTGTAGCGCCACATCTTCTCTTTCTTCCTCGCTGTCCACGATTACCTCTGGTGATGGCGGGCGCTCGGGCTTGGGAGAAGGGCGCTTCTTTTTCTTCTTGGGCGCAATGGCCAAATCCGCCGCCGAGGTCGATGGCCGCGGGCTGGGTGTGCGCGGCACCAGCGCGTCTTGTGATGAGTCTTCCTCGTCCTCGGACTCGATACGCCGCCTCATCCGCTTTTTTGGGGGCGCCCGGGGAGGCGGCGGCGACGGGGACGGGGACGACACGTCCTCCATGGTTGGGGGACGTCGCGCCGCACCGCGTCCGCGCTCGGGGGTGGTTTCGCGCTGCTCCTCTTCCCGACTGGCCATTTCCTTCTCCTATAGGCAGAAAAAGATCATGGAGTCAGTCGAGAAGAAGGACAGCCTAACCGCCCCCTCTGAGTTCGCCACCACCGCCTCCACCGATGCCGCCAACGCGCCTACCACCTTCCCCGTCGAGGCACCCCCGCTTGAGGAGGAGGAAGTGATTATCGAGCAGGACCCAGGTTTTGTAAGCGAAGACGACGAGGACCGCTCAGTACCAACAGAGGATAAAAAGCAAGACCAGGACAACGCAGAGGCAAACGAGGAACAAGTCGGGCGGGGGGACGAAAGGCATGGCGACTACCTAGATGTGGGAGACGACGTGCTGTTGAAGCATCTGCAGCGCCAGTGCGCCATTATCTGCGACGCGTTGCAAGAGCGCAGCGATGTGCCCCTCGCCATAGCGGATGTCAGCCTTGCCTACGAACGCCACCTATTCTCACCGCGCGTACCCCCCAAACGCCAAGAAAACGGCACATGCGAGCCCAACCCGCGCCTCAACTTCTACCCCGTATTTGCCGTGCCAGAGGTGCTTGCCACCTATCACATCTTTTTCCAAAACTGCAAGATACCCCTATCCTGCCGTGCCAACCGCAGCCGAGCGGACAAGCAGCTGGCCTTGCGGCAGGGCGCTGTCATACCTGATATCGCCTCGCTCAACGAAGTGCCAAAAATCTTTGAGGGTCTTGGACGCGACGAGAAGCGCGCGGCAAACGCTCTGCAACAGGAAAACAGCGAAAATGAAAGTCACTCTGGAGTGTTGGTGGAACTCGAGGGTGACAACGCGCGCCTAGCCGTACTAAAACGCAGCATCGAGGTCACCCACTTTGCCTACCCGGCACTTAACCTACCCCCCAAGGTCATGAGCACAGTCATGAGTGAGCTGATCGTGCGCCGTGCGCAGCCCCTGGAGAGGGATGCAAATTTGCAAGAACAAACAGAGGAGGGCCTACCCGCAGTTGGCGACGAGCAGCTAGCGCGCTGGCTTCAAACGCGCGAGCCTGCCGACTTGGAGGAGCGACGCAAACTAATGATGGCCGCAGTGCTCGTTACCGTGGAGCTTGAGTGCATGCAGCGGTTCTTTGCTGACCCGGAGATGCAGCGCAAGCTAGAGGAAACATTGCACTACACCTTTCGACAGGGCTACGTACGCCAGGCCTGCAAGATCTCCAACGTGGAGCTCTGCAACCTGGTCTCCTACCTTGGAATTTTGCACGAAAACCGCCTTGGGCAAAACGTGCTTCATTCCACGCTCAAGGGCGAGGCGCGCCGCGACTACGTCCGCGACTGCGTTTACTTATTTCTATGCTACACCTGGCAGACGGCCATGGGCGTTTGGCAGCAGTGCTTGGAGGAGTGCAACCTCAAGGAGCTGCAGAAACTGCTAAAGCAAAACTTGAAGGACCTATGGACGGCCTTCAACGAGCGCTCCGTGGCCGCGCACCTGGCGGACATCATTTTCCCCGAACGCCTGCTTAAAACCCTGCAACAGGGTCTGCCAGACTTCACCAGTCAAAGCATGTTGCAGAACTTTAGGAACTTTATCCTAGAGCGCTCAGGAATCTTGCCCGCCACCTGCTGTGCACTTCCTAGCGACTTTGTGCCCATTAAGTACCGCGAATGCCCTCCGCCGCTTTGGGGCCACTGCTACCTTCTGCAGCTAGCCAACTACCTTGCCTACCACTCTGACATAATGGAAGACGTGAGCGGTGACGGTCTACTGGAGTGTCACTGTCGCTGCAACCTATGCACCCCGCACCGCTCCCTGGTTTGCAATTCGCAGCTGCTTAACGAAAGTCAAATTATCGGTACCTTTGAGCTGCAGGGTCCCTCGCCTGACGAAAAGTCCGCGGCTCCGGGGTTGAAACTCACTCCGGGGCTGTGGACGTCGGCTTACCTTCGCAAATTTGTACCTGAGGACTACCACGCCCACGAGATTAGGTTCTACGAAGACCAATCCCGCCCGCCTAATGCGGAGCTTACCGCCTGCGTCATTACCCAGGGCCACATTCTTGGCCAATTGCAAGCCATCAACAAAGCCCGCCAAGAGTTTCTGCTACGAAAGGGACGGGGGGTTTACTTGGACCCCCAGTCCGGCGAGGAGCTCAACCCAATCCCCCCGCCGCCGCAGCCCTATCAGCAGCAGCCGCGGGCCCTTGCTTCCCAGGATGGCACCCAAAAAGAAGCTGCAGCTGCCGCCGCCACCCACGGACGAGGAGGAATACTGGGACAGTCAGGCAGAGGAGGTTTTGGACGAGGAGGAGGAGGACATGATGGAAGACTGGGAGAGCCTAGACGAGGAAGCTTCCGAGGTCGAAGAGGTGTCAGACGAAACACCGTCACCCTCGGTCGCATTCCCCTCGCCGGCGCCCCAGAAATCGGCAACCGGTTCCAGCATGGCTACAACCTCCGCTCCTCAGGCGCCGCCGGCACTGCCCGTTCGCCGACCCAACCGTAGATGGGACACCACTGGAACCAGGGCCGGTAAGTCCAAGCAGCCGCCGCCGTTAGCCCAAGAGCAACAACAGCGCCAAGGCTACCGCTCATGGCGCGGGCACAAGAACGCCATAGTTGCTTGCTTGCAAGACTGTGGGGGCAACATCTCCTTCGCCCGCCGCTTTCTTCTCTACCATCACGGCGTGGCCTTCCCCCGTAACATCCTGCATTACTACCGTCATCTCTACAGCCCATACTGCACCGGCGGCAGCGGCAGCAACAGCAGCGGCCACACAGAAGCAAAGGCGACCGGATAGCAAGACTCTGACAAAGCCCAAGAAATCCACAGCGGCGGCAGCAGCAGGAGGAGGAGCGCTGCGTCTGGCGCCCAACGAACCCGTATCGACCCGCGAGCTTAGAAACAGGATTTTTCCCACTCTGTATGCTATATTTCAACAGAGCAGGGGCCAAGAACAAGAGCTGAAAATAAAAAACAGGTCTCTGCGATCCCTCACCCGCAGCTGCCTGTATCACAAAAGCGAAGATCAGCTTCGGCGCACGCTGGAAGACGCGGAGGCTCTCTTCAGTAAATACTGCGCGCTGACTCTTAAGGACTAGTTTCGCGCCCTTTCTCAAATTTAAGCGCGAAAACTACGTCATCTCCAGCGGCCACACCCGGCGCCAGCACCTGTTGTCAGCGCCATTATGAGCAAGGAAATTCCCACGCCCTACATGTGGAGTTACCAGCCACAAATGGGACTTGCGGCTGGAGCTGCCCAAGACTACTCAACCCGAATAAACTACATGAGCGCGGGACCCCACATGATATCCCGGGTCAACGGAATACGCGCCCACCGAAACCGAATTCTCCTGGAACAGGCGGCTATTACCACCACACCTCGTAATAACCTTAATCCCCGTAGTTGGCCCGCTGCCCTGGTGTACCAGGAAAGTCCCGCTCCCACCACTGTGGTACTTCCCAGAGACGCCCAGGCCGAAGTTCAGATGACTAACTCAGGGGCGCAGCTTGCGGGCGGCTTTCGTCACAGGGTGCGGTCGCCCGGGCAGGGTATAACTCACCTGACAATCAGAGGGCGAGGTATTCAGCTCAACGACGAGTCGGTGAGCTCCTCGCTTGGTCTCCGTCCGGACGGGACATTTCAGATCGGCGGCGCCGGCCGCTCTTCATTCACGCCTCGTCAGGCAATCCTAACTCTGCAGACCTCGTCCTCTGAGCCGCGCTCTGGAGGCATTGGAACTCTGCAATTTATTGAGGAGTTTGTGCCATCGGTCTACTTTAACCCCTTCTCGGGACCTCCCGGCCACTATCCGGATCAATTTATTCCTAACTTTGACGCGGTAAAGGACTCGGCGGACGGCTACGACTGAATGTTAAGTGGAGAGGCAGAGCAACTGCGCCTGAAACACCTGGTCCACTGTCGCCGCCACAAGTGCTTTGCCCGCGACTCCGGTGAGTTTTGCTACTTTGAATTGCCCGAGGATCATATCGAGGGCCCGGCGCACGGCGTCCGGCTTACCGCCCAGGGAGAGCTTGCCCGTAGCCTGATTCGGGAGTTTACCCAGCGCCCCCTGCTAGTTGAGCGGGACAGGGGACCCTGTGTTCTCACTGTGATTTGCAACTGTCCTAACCCTGGATTACATCAAGATCCTCTAGTTAATGTCAGGTCGCCTAAGTCGATTAACTAGAGTACCCGGGGATCTTATTCCCTTTAACTAATAAAAAAAAATAATAAAGCATCACTTACTTAAAATCAGTTAGCAAATTTCTGTCCAGTTTATTCAGCAGCACCTCCTTGCCCTCCTCCCAGCTCTGGTATTGCAGCTTCCTCCTGGCTGCAAACTTTCTCCACAATCTAAATGGAATGTCAGTTTCCTCCTGTTCCTGTCCATCCGCACCCACTATCTTCATGTTGTTGCAGATGAAGCGCGCAAGACCGTCTGAAGATACCTTCAACCCCGTGTATCCATATGACACGGAAACCGGTCCTCCAACTGTGCCTTTTCTTACTCCTCCCTTTGTATCCCCCAATGGGTTTCAAGAGAGTCCCCCTGGGGTACTCTCTTTGCGCCTATCCGAACCTCTAGTTACCTCCAATGGCATGCTTGCGCTCAAAATGGGCAACGGCCTCTCTCTGGACGAGGCCGGCAACCTTACCTCCCAAAATGTAACCACTGTGAGCCCACCTCTCAAAAAAACCAAGTCAAACATAAACCTGGAAATATCTGCACCCCTCACAGTTACCTCAGAAGCCCTAACTGTGGCTGCCGCCGCACCTCTAATGGTCGCGGGCAACACACTCACCATGCAATCACAGGCCCCGCTAACCGTGCACGACTCCAAACTTAGCATTGCCACCCAAGGACCCCTCACAGTGTCAGAAGGAAAGCTAGCCCTGCAAACATCAGGCCCCCTCACCACCACCGATAGCAGTACCCTTACTATCACTGCCTCACCCCCTCTAACTACTGCCACTGGTAGCTTGGGCATTGACTTGAAAGAGCCCATTTATACACAAAATGGAAAACTAGGACTAAAGTACGGGGCTCCTTTGCATGTAACAGACGACCTAAACACTTTGACCGTAGCAACTGGTCCAGGTGTGACTATTAATAATACTTCCTTGCAAACTAAAGTTACTGGAGCCTTGGGTTTTGATTCACAAGGCAATATGCAACTTAATGTAGCAGGAGGACTAAGGATTGATTCTCAAAACAGACGCCTTATACTTGATGTTAGTTATCCGTTTGATGCTCAAAACCAACTAAATCTAAGACTAGGACAGGGCCCTCTTTTTATAAACTCAGCCCACAACTTGGATATTAACTACAACAAAGGCCTTTACTTGTTTACAGCTTCAAACAATTCCAAAAAGCTTGAGGTTAACCTAAGCACTGCCAAGGGGTTGATGTTTGACGCTACAGCCATAGCCATTAATGCAGGAGATGGGCTTGAATTTGGTTCACCTAATGCACCAAACACAAATCCCCTCAAAACAAAAATTGGCCATGGCCTAGAATTTGATTCAAACAAGGCTATGGTTCCTAAACTAGGAACTGGCCTTAGTTTTGACAGCACAGGTGCCATTACAGTAGGAAACAAAAATAATGATAAGCTAACTTTGTGGACCACACCAGCTCCATCTCCTAACTGTAGACTAAATGCAGAGAAAGATGCTAAACTCACTTTGGTCTTAACAAAATGTGGCAGTCAAATACTTGCTACAGTTTCAGTTTTGGCTGTTAAAGGCAGTTTGGCTCCAATATCTGGAACAGTTCAAAGTGCTCATCTTATTATAAGATTTGACGAAAATGGAGTGCTACTAAACAATTCCTTCCTGGACCCAGAATATTGGAACTTTAGAAATGGAGATCTTACTGAAGGCACAGCCTATACAAACGCTGTTGGATTTATGCCTAACCTATCAGCTTATCCAAAATCTCACGGTAAAACTGCCAAAAGTAACATTGTCAGTCAAGTTTACTTAAACGGAGACAAAACTAAACCTGTAACACTAACCATTACACTAAACGGTACACAGGAAACAGGAGACACAACTCCAAGTGCATACTCTATGTCATTTTCATGGGACTGGTCTGGCCACAACTACATTAATGAAATATTTGCCACATCCTCTTACACTTTTTCATACATTGCCCAAGAATAAAGAATCGTTTGTGTTATGTTTCAACGTGTTTATTTTTCAATTGCAGAAAATTTCAAGTCATTTTTCATTCAGTAGTATAGCCCCACCACCACATAGCTTATACAGATCACCGTACCTTAATCAAACTCACAGAACCCTAGTATTCAACCTGCCACCTCCCTCCCAACACACAGAGTACACAGTCCTTTCTCCCCGGCTGGCCTTAAAAAGCATCATATCATGGGTAACAGACATATTCTTAGGTGTTATATTCCACACGGTTTCCTGTCGAGCCAAACGCTCATCAGTGATATTAATAAACTCCCCGGGCAGCTCACTTAAGTTCATGTCGCTGTCCAGCTGCTGAGCCACAGGCTGCTGTCCAACTTGCGGTTGCTTAACGGGCGGCGAAGGAGAAGTCCACGCCTACATGGGGGTAGAGTCATAATCGTGCATCAGGATAGGGCGGTGGTGCTGCAGCAGCGCGCGAATAAACTGCTGCCGCCGCCGCTCCGTCCTGCAGGAATACAACATGGCAGTGGTCTCCTCAGCGATGATTCGCACCGCCCGCAGCATAAGGCGCCTTGTCCTCCGGGCACAGCAGCGCACCCTGATCTCACTTAAATCAGCACAGTAACTGCAGCACAGCACCACAATATTGTTCAAAATCCCACAGTGCAAGGCGCTGTATCCAAAGCTCATGGCGGGGACCACAGAACCCACGTGGCCATCATACCACAAGCGCAGGTAGATTAAGTGGCGACCCCTCATAAACACGCTGGACATAAACATTACCTCTTTTGGCATGTTGTAATTCACCACCTCCCGGTACCATATAAACCTCTGATTAAACATGGCGCCATCCACCACCATCCTAAACCAGCTGGCCAAAACCTGCCCGCCGGCTATACACTGCAGGGAACCGGGACTGGAACAATGACAGTGGAGAGCCCAGGACTCGTAACCATGGATCATCATGCTCGTCATGATATCAATGTTGGCACAACACAGGCACACGTGCATACACTTCCTCAGGATTACAAGCTCCTCCCGCGTTAGAACCATATCCCAGGGAACAACCCATTCCTGAATCAGCGTAAATCCCACACTGCAGGGAAGACCTCGCACGTAACTCACGTTGTGCATTGTCAAAGTGTTACATTCGGGCAGCAGCGGATGATCCTCCAGTATGGTAGCGCGGGTTTCTGTCTCAAAAGGAGGTAGACGATCCCTACTGTACGGAGTGCGCCGAGACAACCGAGATCGTGTTGGTCGTAGTGTCATGCCAAATGGAACGCCGGACGTAGTCATATTTCCTGAAGCAAAACCAGGTGCGGGCGTGACAAACAGATCTGCGTCTCCGGTCTCGCCGCTTAGATCGCTCTGTGTAGTAGTTGTAGTATATCCACTCTCTCAAAGCATCCAGGCGCCCCCTGGCTTCGGGTTCTATGTAAACTCCTTCATGCGCCGCTGCCCTGATAACATCCACCACCGCAGAATAAGCCACACCCAGCCAACCTACACATTCGTTCTGCGAGTCACACACGGGAGGAGCGGGAAGAGCTGGAAGAACCATGTTTTTTTTTTATTCCAAAAGATTATCCAAAACCTCAAAATGAAGATCTATTAAGTGAACGCGCTCCCCTCCGGTGGCGTGGTCAAACTCTACAGCCAAAGAACAGATAATGGCATTTGTAAGATGTTGCACAATGGCTTCCAAAAGGCAAACGGCCCTCACGTCCAAGTGGACGTAAAGGCTAAACCCTTCAGGGTGAATCTCCTCTATAAACATTCCAGCACCTTCAACCATGCCCAAATAATTCTCATCTCGCCACCTTCTCAATATATCTCTAAGCAAATCCCGAATATTAAGTCCGGCCATTGTAAAAATCTGCTCCAGAGCGCCCTCCACCTTCAGCCTCAAGCAGCGAATCATGATTGCAAAAATTCAGGTTCCTCACAGACCTGTATAAGATTCAAAAGCGGAACATTAACAAAAATACCGCGATCCCGTAGGTCCCTTCGCAGGGCCAGCTGAACATAATCGTGCAGGTCTGCACGGACCAGCGCGGCCACTTCCCCGCCAGGAACCATGACAAAAGAACCCACACTGATTATGACACGCATACTCGGAGCTATGCTAACCAGCGTAGCCCCGATGTAAGCTTGTTGCATGGGCGGCGATATAAAATGCAAGGTGCTGCTCAAAAAATCAGGCAAAGCCTCGCGCAAAAAAGAAAGCACATCGTAGTCATGCTCATGCAGATAAAGGCAGGTAAGCTCCGGAACCACCACAGAAAAAGACACCATTTTTCTCTCAAACATGTCTGCGGGTTTCTGCATAAACACAAAATAAAATAACAAAAAAACATTTAAACATTAGAAGCCTGTCTTACAACAGGAAAAACAACCCTTATAAGCATAAGACGGACTACGGCCATGCCGGCGTGACCGTAAAAAAACTGGTCACCGTGATTAAAAAGCACCACCGACAGCTCCTCGGTCATGTCCGGAGTCATAATGTAAGACTCGGTAAACACATCAGGTTGATTCACATCGGTCAGTGCTAAAAAGCGACCGAAATAGCCCGGGGGAATACATACCCGCAGGCGTAGAGACAACATTACAGCCCCCATAGGAGGTATAACAAAATTAATAGGAGAGAAAAACACATAAACACCTGAAAAACCCTCCTGCCTAGGCAAAATAGCACCCTCCCGCTCCAGAACAACATACAGCGCTTCCACAGCGGCAGCCATAACAGTCAGCCTTACCAGTAAAAAAGAAAACCTATTAAAAAAACACCACTCGACACGGCACCAGCTCAATCAGTCACAGTGTAAAAAAGGGCCAAGTGCAGAGCGAGTATATATAGGACTAAAAAATGACGTAACGGTTAAAGTCCACAAAAAACACCCAGAAAACCGCACGCGAACCTACGCCCAGAAACGAAAGCCAAAAAACCCACAACTTCCTCAAATCGTCACTTCCGTTTTCCCACGTTACGTCACTTCCCATTTTAAGAAAACTACAATTCCCAACACATACAAGTTACTCCGCCCTAAAACCTACGTCACCCGCCCCGTTCCCACGCCCCGCGCCACGTCACAAACTCCACCCCCTCATTATCATATTGGCTTCAATCCAAAATAAGGTATATTATTGATGATG

- EGFP CDS in ADV-EGFP dG: 20-nt gRNA175 hybrid region is underlined.


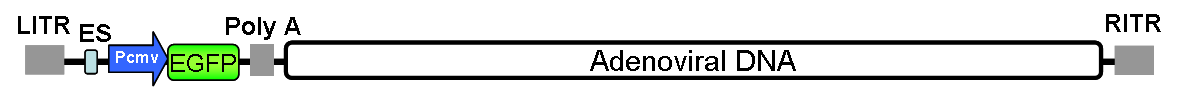


…….**ATGGTGAGCAAGGGCGAGGAGCTGTTCACCGGGGTGGTGCCCATCCTGGTCGAGCTGGACGGCGACGTAAACGGCCACAAGTTCAGCGTGTCCGGCGAGGGCGAGGGCGATGCCACCTACGGCAAGCTGACCCTGAAGTTCATCTGCACCACCGGCAAGCTGCCCGTGCCCTGGCCCACCCTCGTGACCACCCTGACCTACG-CGTGCAGTGCTTCAGCCGCTACCCCGACCACATGAAGCAGCACGACTTCTTCAAGTCCGCCATGCCCGAAGGCTACGTCCAGGAGCGCACCATCTTCTTCAAGGACGACGGCAACTACAAGACCCGCGCCGAGGTGAAGTTCGAGGGCGACACCCTGGTGAACCGCATCGAGCTGAAGGGCATCGACTTCAAGGAGGACGGCAACATCCTGGGGCACAAGCTGGAGTACAACTACAACAGCCACAACGTCTATATCATGGCCGACAAGCAGAAGAACGGCATCAAGGTGAACTTCAAGATCCGCCACAACATCGAGGACGGCAGCGTGCAGCTCGCCGACCACTACCAGCAGAACACCCCCATCGGCGACGGCCCCGTGCTGCTGCCCGACAACCACTACCTGAGCACCCAGTCCGCCCTGAGCAAAGACCCCAACGAGAAGCGCGATCACATGGTCCTGCTGGAGTTCGTGACCGCCGCCGGGATCACTCTCGGCATGGACGAGCTGTACAAGTAA**……

- ADV-DsRed genome 1-4232bp: DsRed ORF is highlighted


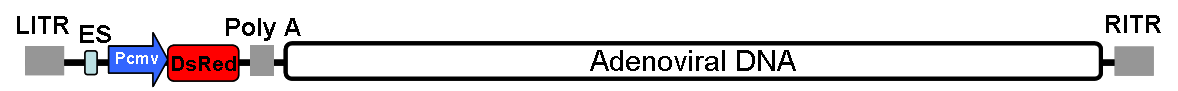


CATCATCAATAATATACCTTATTTTGGATTGAAGCCAATATGATAATGAGGGGGTGGAGTTTGTGACGTGGCGCGGGGCGTGGGAACGGGGCGGGTGACGTAGTAGTGTGGCGGAAGTGTGATGTTGCAAGTGTGGCGGAACACATGTAAGCGACGGATGTGGCAAAAGTGACGTTTTTGGTGTGCGCCGGTGTACACAGGAAGTGACAATTTTCGCGCGGTTTTAGGCGGATGTTGTAGTAAATTTGGGCGTAACCGAGTAAGATTTGGCCATTTTCGCGGGAAAACTGAATAAGAGGAAGTGAAATCTGAATAATTTTGTGTTACTCATAGCGCGTAATACTGTAATAGTAATCAATTACGGGGTCATTAGTTCATAGCCCATATATGGAGTTCCGCGTTACATAACTTACGGTAAATGGCCCGCCTGGCTGACCGCCCAACGACCCCCGCCCATTGACGTCAATAATGACGTATGTTCCCATAGTAACGCCAATAGGGACTTTCCATTGACGTCAATGGGTGGAGTATTTACGGTAAACTGCCCACTTGGCAGTACATCAAGTGTATCATATGCCAAGTACGCCCCCTATTGACGTCAATGACGGTAAATGGCCCGCCTGGCATTATGCCCAGTACATGACCTTATGGGACTTTCCTACTTGGCAGTACATCTACGTATTAGTCATCGCTATTACCATGGTGATGCGGTTTTGGCAGTACATCAATGGGCGTGGATAGCGGTTTGACTCACGGGGATTTCCAAGTCTCCACCCCATTGACGTCAATGGGAGTTTGTTTTGGCACCAAAATCAACGGGACTTTCCAAAATGTCGTAACAACTCCGCCCCATTGACGCAAATGGGCGGTAGGCGTGTACGGTGGGAGGTCTATATAAGCAGAGCTGGTTTAGTGAACCGTCAGATCCGCTAGAGATCTGGTACCGCGGGCCCGGGATCCACCGGTCGCCACCATGGCCTCCTCCGAGAACGTCATCACCGAGTTCATGCGCTTCAAGGTGCGCATGGAGGGCACCGTGAACGGCCACGAGTTCGAGATCGAGGGCGAGGGCGAGGGCCGCCCCTACGAGGGCCACAACACCGTGAAGCTGAAGGTGACCAAGGGCGGCCCCCTGCCCTTCGCCTGGGACATCCTGTCCCCCCAGTTCCAGTACGGCTCCAAGGTGTACGTGAAGCACCCCGCCGACATCCCCGACTACAAGAAGCTGTCCTTCCCCGAGGGCTTCAAGTGGGAGCGCGTGATGAACTTCGAGGACGGCGGCGTGGCGACCGTGACCCAGGACTCCTCCCTGCAGGACGGCTGCTTCATCTACAAGGTGAAGTTCATCGGCGTGAACTTCCCCTCCGACGGCCCCGTGATGCAGAAGAAGACCATGGGCTGGGAGGCCTCCACCGAGCGCCTGTACCCCCGCGACGGCGTGCTGAAGGGCGAGACCCACAAGGCCCTGAAGCTGAAGGACGGCGGCCACTACCTGGTGGAGTTCAAGTCCATCTACATGGCCAAGAAGCCCGTGCAGCTGCCCGGCTACTACTACGTGGACGCCAAGCTGGACATCACCTCCCACAACGAGGACTACACCATCGTGGAGCAGTACGAGCGCACCGAGGGCCGCCACCACCTGTTCCTGTAGCGGCCGCTCGAGCCTAAGCTTCTAGATAAGATATCCGATCCACCGGATCTAGATAACTGATCATAATCAGCCATACCACATTTGTAGAGGTTTTACTTGCTTTAAAAAACCTCCCACACCTCCCCCTGAACCTGAAACATAAAATGAATGCAATTGTTGTTGTTAACTTGTTTATTGCAGCTTATAATGGTTACAAATAAAGCAATAGCATCACAAATTTCACAAATAAAGCATTTTTTTCACTGCATTCTAGTTGTGGTTTGTCCAAACTCATCAATGTATCTTAACGCGGATCTGGGCGTGGTTAAGGGTGGGAAAGAATATATAAGGTGGGGGTCTTATGTAGTTTTGTATCTGTTTTGCAGCAGCCGCCGCCGCCATGAGCACCAACTCGTTTGATGGAAGCATTGTGAGCTCATATTTGACAACGCGCATGCCCCCATGGGCCGGGGTGCGTCAGAATGTGATGGGCTCCAGCATTGATGGTCGCCCCGTCCTGCCCGCAAACTCTACTACCTTGACCTACGAGACCGTGTCTGGAACGCCGTTGGAGACTGCAGCCTCCGCCGCCGCTTCAGCCGCTGCAGCCACCGCCCGCGGGATTGTGACTGACTTTGCTTTCCTGAGCCCGCTTGCAAGCAGTGCAGCTTCCCGTTCATCCGCCCGCGATGACAAGTTGACGGCTCTTTTGGCACAATTGGATTCTTTGACCCGGGAACTTAATGTCGTTTCTCAGCAGCTGTTGGATCTGCGCCAGCAGGTTTCTGCCCTGAAGGCTTCCTCCCCTCCCAATGCGGTTTAAAACATAAATAAAAAACCAGACTCTGTTTGGATTTGGATCAAGCAAGTGTCTTGCTGTCTTTATTTAGGGGTTTTGCGCGCGCGGTAGGCCCGGGACCAGCGGTCTCGGTCGTTGAGGGTCCTGTGTATTTTTTCCAGGACGTGGTAAAGGTGACTCTGGATGTTCAGATACATGGGCATAAGCCCGTCTCTGGGGTGGAGGTAGCACCACTGCAGAGCTTCATGCTGCGGGGTGGTGTTGTAGATGATCCAGTCGTAGCAGGAGCGCTGGGCGTGGTGCCTAAAAATGTCTTTCAGTAGCAAGCTGATTGCCAGGGGCAGGCCCTTGGTGTAAGTGTTTACAAAGCGGTTAAGCTGGGATGGGTGCATACGTGGGGATATGAGATGCATCTTGGACTGTATTTTTAGGTTGGCTATGTTCCCAGCCATATCCCTCCGGGGATTCATGTTGTGCAGAACCACCAGCACAGTGTATCCGGTGCACTTGGGAAATTTGTCATGTAGCTTAGAAGGAAATGCGTGGAAGAACTTGGAGACGCCCTTGTGACCTCCAAGATTTTCCATGCATTCGTCCATAATGATGGCAATGGGCCCACGGGCGGCGGCCTGGGCGAAGATATTTCTGGGATCACTAACGTCATAGTTGTGTTCCAGGATGAGATCGTCATAGGCCATTTTTACAAAGCGCGGGCGGAGGGTGCCAGACTGCGGTATAATGGTTCCATCCGGCCCAGGGGCGTAGTTACCCTCACAGATTTGCATTTCCCACGCTTTGAGTTCAGATGGGGGGATCATGTCTACCTGCGGGGCGATGAAGAAAACGGTTTCCGGGGTAGGGGAGATCAGCTGGGAAGAAAGCAGGTTCCTGAGCAGCTGCGACTTACCGCAGCCGGTGGGCCCGTAAATCACACCTATTACCGGCTGCAACTGGTAGTTAAGAGAGCTGCAGCTGCCGTCATCCCTGAGCAGGGGGGCCACTTCGTTAAGCATGTCCCTGACTCGCATGTTTTCCCTGACCAAATCCGCCAGAAGGCGCTCGCCGCCCAGCGATAGCAGTTCTTGCAAGGAAGCAAAGTTTTTCAACGGTTTGAGACCGTCCGCCGTAGGCATGCTTTTGAGCGTTTGACCAAGCAGTTCCAGGCGGTCCCACAGCTCGGTCACCTGCTCTACGGCATCTCGATCCAGCATATCTCCTCGTTTCGCGGGTTGGGGCGGCTTTCGCTGTACGGCAGTAGTCGGTGCTCGTCCAGACGGGCCAGGGTCATGTCTTTCCACGGGCGCAGGGTCCTCGTCAGCGTAGTCTGGGTCACGGTGAAGGGGTGCGCTCCGGGCTGCGCGCTGGCCAGGGTGCGCTTGAGGCTGGTCCTGCTGGTGCTGAAGCGCTGCCGGTCTTCGCCCTGCGCGTCGGCCAGGTAGCATTTGACCATGGTGTCATAGTCCAGCCCCTCCGCGGCGTGGCCCTTGGCGCGCAGCTTGCCCTTGGAGGAGGCGCCGCACGAGGGGCAGTGCAGACTTTTGAGGGCGTAGAGCTTGGGCGCGAGAAATACCGATTCCGGGGAGTAGGCATCCGCGCCGCAGGCCCCGCAGACGGTCTCGCATTCCACGAGCCAGGTGAGCTCTGGCCGTTCGGGGTCAAAAACCAGGTTTCCCCCATGCTTTTTGATGCGTTTCTTACCTCTGGTTTCCATGAGCCGGTGTCCACGCTCGGTGACGAAAAGGCTGTCCGTGTCCCCGTATACAGACTTGAGAGGCCTGTCCTCGAGCGGTGTTCCGCGGTCCT……

- HSV1-EGFP：EGFP CDS （highlight）was inserted into TK ORF.

ATGGCTTCGTACCCCGGCCATCAACACGCGTCTGCGTTCGACCAGGCTGCGCGTTCTCGCGGCCATAGCAACCGACGTACGATGGTGAGCAAGGGCGAGGAGCTGTTCACCGGGGTGGTGCCCATCCTGGTCGAGCTGGACGGCGACGTAAACGGCCACAAGTTCAGCGTGTCCGGCGAGGGCGAGGGCGATGCCACCTACGGCAAGCTGACCCTGAAGTTCATCTGCACCACCGGCAAGCTGCCCGTGCCCTGGCCCACCCTCGTGACCACCCTGACCTACGGCGTGCAGTGCTTCAGCCGCTACCCCGACCACATGAAGCAGCACGACTTCTTCAAGTCCGCCATGCCCGAAGGCTACGTCCAGGAGCGCACCATCTTCTTCAAGGACGACGGCAACTACAAGACCCGCGCCGAGGTGAAGTTCGAGGGCGACACCCTGGTGAACCGCATCGAGCTGAAGGGCATCGACTTCAAGGAGGACGGCAACATCCTGGGGCACAAGCTGGAGTACAACTACAACAGCCACAACGTCTATATCATGGCCGACAAGCAGAAGAACGGCATCAAGGTGAACTTCAAGATCCGCCACAACATCGAGGACGGCAGCGTGCAGCTCGCCGACCACTACCAGCAGAACACCCCCATCGGCGACGGCCCCGTGCTGCTGCCCGACAACCACTACCTGAGCACCCAGTCCGCCCTGAGCAAAGACCCCAACGAGAAGCGCGATCACATGGTCCTGCTGGAGTTCGTGACCGCCGCCGGGATCACTCTCGGCATGGACGAGCTGTACAAGTAACGTACGGCGTTGCGCCCTCGCCGGCAGCAAGAAGCCACGGAAGTCCGCCCGGAGCAGAAAATGCCCACGCTACTGCGGGTTTATATAGACGGTCCCCACGGGATGGGGAAAACCACCACCACGCAACTGCTGGTGGCCCTGGGTTCGCGCGACGATATCGTCTACGTACCCGAGCCGATGACTTACTGGCGGGTGCTGGGGGCTTCCGAGACAATCGCGAACATCTACACCACACAACACCGCCTCGACCAGGGTGAGATATCGGCCGGGGACGCGGCGGTGGTAATGACAAGCGCCCAGATAACAATGGGCATGCCTTATGCCGTGACCGACGCCGTTCTGGCTCCTCATATCGGGGGGGAGGCTGGGAGCTCACATGCCCCGCCCCCGGCCCTCACCCTCATCTTCGACCGCCATCCCATCGCCGCCCTCCTGTGCTACCCGGCCGCGCGGTACCTTATGGGCAGCATGACCCCCCAGGCCGTGCTGGCGTTCGTGGCCCTCATCCCGCCGACCTTGCCCGGCACCAACATCGTGCTTGGGGCCCTTCCGGAGGACAGACACATCGACCGCCTGGCCAAACGCCAGCGCCCCGGCGAGCGGCTGGACCTGGCTATGCTGGCTGCGATTCGCCGCGTTTACGGGCTACTTGCCAATACGGTGCGGTATCTGCAGTGCGGCGGGTCGTGGCGGGAGGACTGGGGACAGCTTTCGGGGACGGCCGTGCCGCCCCAGGGTGCCGAGCCCCAGAGCAACGCGGGCCCACGACCCCATATCGGGGACACGTTATTTACCCTGTTTCGGGCCCCCGAGTTGCTGGCCCCCAACGGCGACCTGTATAACGTGTTTGCCTGGGCCTTGGACGTCTTGGCCAAACGCCTCCGTTCCATGCACGTCTTTATCCTGGATTACGACCAATCGCCCGCCGGCTGCCGGGACGCCCTGCTGCAACTTACCTCCGGGATGGTCCAGACCCACGTCACCACCCCCGGCTCCATACCGACGATATGCGACCTGGCGCGCACGTTTGCCCGGGAGATGGGGGAGGCTAACTGA
